# Hybrid Minigene Assay: An Efficient Tool to Characterize mRNA Splicing Profiles of *NF1* Variants

**DOI:** 10.3390/cancers13050999

**Published:** 2021-02-27

**Authors:** Valeria Morbidoni, Elisa Baschiera, Monica Forzan, Valentina Fumini, Dario Seif Ali, Gianpietro Giorgi, Lisa Buson, Maria Andrea Desbats, Matteo Cassina, Maurizio Clementi, Leonardo Salviati, Eva Trevisson

**Affiliations:** 1 Clinical Genetics Unit, Department of Women’s and Children’s Health, University of Padova, 35128 Padova, Italy; v.morbidoni@irpcds.org (V.M.); elisa.baschiera@studenti.unipd.it (E.B.); monica.forzan@aopd.veneto.it (M.F.); valentina.fumini@studenti.unipd.it (V.F.); dario.seifali@studenti.unipd.it (D.S.A.); gianpietrogiorgi@gmail.com (G.G.); lisa.buson@unipd.it (L.B.); mariaandrea.desbats@unipd.it (M.A.D.); matteo.cassina@unipd.it (M.C.); maurizio.clementi@unipd.it (M.C.); leonardo.salviati@unipd.it (L.S.); 2 Istituto di Ricerca Pediatrica—IRP, Fondazione Città della Speranza, 35127 Padova, Italy

**Keywords:** neurofibromatosis type 1, *NF1*, splicing, minigene, hypomorphic, leaky splicing

## Abstract

**Simple Summary:**

Heterozygous inactivating mutations in *NF1* cause neurofibromatosis type 1, an autosomal dominant neurocutaneous disorder that shows variable clinical expressivity. Mutational spectrum is wide with a proportion of variants acting on splicing. Molecular diagnosis mostly relies on a combination of *NF1* sequencing with multiplex ligation-dependent probe amplification (MLPA) analysis, but validation may be difficult. We employed a minigene system to examine a series of *NF1* variants identified in patients. Our system allowed to assess the effect(s) on splicing for all the 29 variants we investigated, revealing multiple mechanisms of splicing alterations for some of them. One *NF1* variant identified in a patient with NF1 allowed the production of residual levels of wild-type transcript. Our in vitro minigene system proved to be a fast and reliable method, which may be easily applied to validate *NF1* variants, define their molecular mechanism(s), and detect hypomorphic effects of the variations.

**Abstract:**

Neurofibromatosis type 1 (NF1) is caused by heterozygous loss of function mutations in the *NF1* gene. Although patients are diagnosed according to clinical criteria and few genotype-phenotype correlations are known, molecular analysis remains important. *NF1* displays allelic heterogeneity, with a high proportion of variants affecting splicing, including deep intronic alleles and changes outside the canonical splice sites, making validation problematic. Next Generation Sequencing (NGS) technologies integrated with multiplex ligation-dependent probe amplification (MLPA) have largely overcome RNA-based techniques but do not detect splicing defects. A rapid minigene-based system was set up to test the effects of *NF1* variants on splicing. We investigated 29 intronic and exonic *NF1* variants identified in patients during the diagnostic process. The minigene assay showed the coexistence of multiple mechanisms of splicing alterations for seven variants. A leaky effect on splicing was documented in one *de novo* substitution detected in a sporadic patient with a specific phenotype without neurofibromas. Our splicing assay proved to be a reliable and fast method to validate novel *NF1* variants potentially affecting splicing and to detect hypomorphic effects that might have phenotypic consequences, avoiding the requirement of patient’s RNA.

## 1. Introduction

Neurofibromatosis type 1 (NF1) (MIM #162200) represents one of the most frequent autosomal dominant conditions [1] and it is characterized by cutaneous manifestations that are shared by most patients (café-au-lait spots (CALs) and skinfold freckling (SF)), who however may show a wide clinical heterogeneity, even within the same family. The clinical hallmark of NF1 is the development of neurofibromas: the dermal ones usually appear after puberty, while plexiform neurofibromas are typically congenital. Complications can affect virtually any organ and system and comprise learning disabilities, skeletal dysplasia, scoliosis, vascular stenosis, optic pathway glioma, and other tumors [2]. 

NF1 is caused by heterozygous inactivating mutations of the *NF1* gene, with approximately half of the cases being sporadic. This gene spans about 350 kb on 17q11.2 [3,4] and encodes neurofibromin, which inhibits the RAS pathway through GRD, a specific GTPase activation domain [5,6].

Previous studies have shown that the *NF1* gene displays a complex splicing pattern in order to generate a great diversity of transcripts: 5 *inframe* exons undergo alternative splicing (exon 9a, exon 10a-2, exon 23a, exon 43, and exon 48a) [4]. Alternative splicing also includes the skipping of some exons (such 29 or 30 or both) or the N-isoform with additional 241 base pairs (bp) of intron 10c and several additional shorter transcripts whose physiological significance remains unclear [7,8]. Total RNA extracted from peripheral leukocytes contains isoform 1 (NM_001042492.3), which is the longest transcript with an open reading frame of 8520 kb, and isoform 2 (NM_000267.3), lacking exon 23a [9].

Patients are diagnosed clinically according to well-established international criteria [10], which are now being updated. Given the existence of non-allelic heterogeneity [11] and the discovery of some genotype-phenotype correlations, the inclusion of a pathogenic variant in *NF1* among the diagnostic criteria has been advocated since 2008 [12]. Molecular analysis remains crucial in sporadic cases (to confirm the diagnosis in young children still not fulfilling the NIH criteria and to differentiate NF1 from Legius syndrome, which is caused by mutations in the *SPRED1* gene), in adults requesting a prenatal/preimplantation diagnosis, or in patients presenting with spinal NF1 that may have very limited skin involvement [13], and in other atypical cases. In fact, about 5% of patients still do not have a definitive clinical diagnosis by the age of eight [14].

NF1 is characterized by a wide mutational spectrum, with pathogenic variants spread over the entire gene and including single nucleotide substitutions, small *inframe* and *frameshift* insertions/deletions, large microdeletions, and single or multi exons duplications/deletions [15,16]. So far, a handful of genotype-phenotype correlations have been recognized in NF1: (i) a more severe clinical course in patients harboring whole gene deletions [17,18]; (ii) the absence of neurofibromas in patients harboring the *inframe* c.2970-2972delAAT deletion [19,20], a missense variant at codon 1809 [21,22], or the Arg1038Gly variant [23]; (iii) a more severe phenotype with higher frequency of spinal and deep neurofibromas in patients harboring a missense variant affecting codons 844–848 [24]; (iv) three specific recurrent non-truncating *NF1* hotspots (codons p.Met1149, p.Arg1276, and p.Lys1423) have been associated with Noonan-like features with specific phenotypes: p.Met1149 was associated with a mild phenotype characterized by pigmentary manifestations; p.Arg1276 and p.Lys1423 were found in patients with a higher prevalence of pulmonary stenosis and other cardiovascular anomalies; p.Arg1276 was associated with a higher rate of plexiform and symptomatic spinal neurofibromas [25].

Given the size and complexity of the *NF1* gene (with a considerable number of pseudogenes) and the wide allelic heterogeneity, high sensitivity in genetic testing has been classically achieved through a comprehensive analysis consisting of a multi-step approach, including an RNA-based molecular assay and methods to detect copy number changes [26]. Although these methods can achieve a higher detection rate [27], patient’s RNA is not always available and cultivation of lymphocytes before RNA extraction [28] or rapid processing of blood samples after venipuncture [29] are necessary to prevent illegitimate splicing. However, recently, most laboratories have set up high-throughput deep sequencing protocols for *NF1* mutational screening from genomic DNA [30,31,32,33], using cDNA-based methods for second-level analyses. Although techniques using genomic DNA are faster and cheaper, they often pose the problem of interpreting the pathogenicity of novel variants. In fact, most *NF1* mutations are private, and, except for large deletions or truncating alleles, the unequivocal distinction between a pathogenic and a benign variant is still challenging, given the lack of information about most domains of neurofibromin and the difficulty of setting up functional assays. Moreover, a high proportion of point mutations causes missplicing with pseudoexons inclusion or exon skipping [15,34,35] that, unlike cDNA analysis, cannot be detected through genomic DNA sequencing. Of note, beyond typical splice variants in the *NF1* gene, also coding region variants (missense, nonsense, and silent mutations) [36], intronic changes outside the canonical AG/GT nucleotides of the 5′ or 3′ splice sites, or deep intronic variants [32] have been found to affect splicing. 

In this work, we describe a valuable system based on a well-established hybrid minigene to test the effects of point variants on *NF1* splicing; this assay does not require patient’s RNA and allows to analyze splicing of transcripts expressed from a single allele.

## 2. Results

Since a significant proportion of *NF1* variants affects splicing, we used an in vitro minigene assay to examine a series of germline variants in the *NF1* gene identified in our cohort of patients with a clinical diagnosis/suspicion of neurofibromatosis type 1 (Table 1 and Supplementary Table S1 for detailed clinical features). 

We analyzed as controls a set of 10 *NF1* benign variants (8 intronic and 2 exonic) that were identified during the diagnostic procedures in patients with a *NF1* pathogenic variant, that do not segregate with the phenotype, or exhibit an allele frequency incompatible with the disease prevalence. Then we investigated the effects on splicing of 19 *NF1* variants, including 14 intronic changes (5 affecting the canonical splice sites) and 5 exonic substitutions. 

At first, we performed an in silico analysis in order to predict their possible effects on splicing (Table 2 and Table 3). Remarkably, the bioinformatic analyses predicted a benign effect only for two (c.1393-82dupT and c.7259-17C > T) out of the 10 variants used as controls; a missplicing effect was predicted for the synonym variant c.5694G > A p.(Glu1898=), whereas conflicting results were obtained for the remaining 7 changes. All 5 variations involving the 5′ or 3′ GT/AG canonical splice sites of *NF1* were correctly classified as splicing affecting variants by all the predicting software tools. For the remaining 14 non-canonical variants (including exonic changes or those involving intronic residues outside the conserved dinucleotides at the splice site boundaries), concordant results were obtained for 12 substitutions, whereas in two cases (c.7250_7252delACT and c.278G > A) different programs yielded conflicting results. Therefore, we decided to examine all these variants using a classical splicing minigene assay, which we previously successfully employed to analyze *ASL*, *CFTR*, and *SF3B4* variants [37,38,39,40] and that has been already used by other groups for validating *NF1* changes [8,36].

### 2.1. NF1 Benign Variants

**Figure 1:**
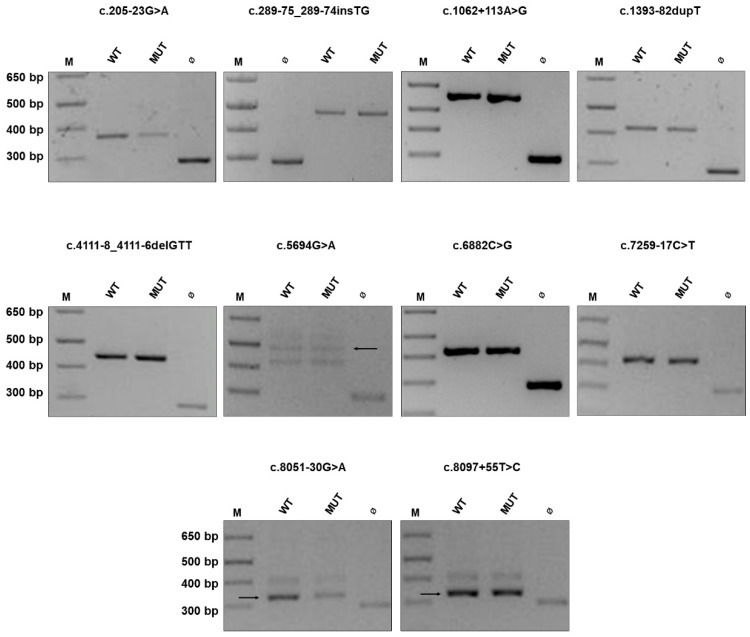
*NF1* variants that do not affect gene splicing. Agarose gel electrophoresis of RT-PCR products obtained after transfection of HEK293 cells with minigene constructs harboring intronic and exonic *NF1* variants that do not affect gene splicing (c.205-23G > A, c.289-75_289-74insTG, c.1062 + 113A > G, c.1393-82dupT, c.4111-8_4111-6delGTT, c.5694G > A, c.6882C > G, c.7259-17C > T, c.8051-30G > A, c.8097 + 55T > C). Cells were transfected with constructs harboring the indicated variants (MUT) or with the corresponding wild-type ones (WT), while the empty vector (Ø) was used as internal control where indicated. If more than one band is visible in gel lanes, a black arrow points at the correct one. M, 1 Kb Plus DNA Ladder (ThermoFisher Scientific).

For all the benign variants we examined, the expression of the mutant minigene construct did not affect transcript maturation: the same splicing pattern as that obtained with the wild-type plasmid was observed (Figure 1), confirming the reliability of our minigene in vitro assay. Remarkably, for some variants, our results differ from bioinformatic data and previous findings. Particularly, the c.1062 + 113A > G transition, which is predicted to affect splicing (Table 2), was previously reported in one adult Italian patient who did not fulfill diagnostic criteria (he presented scoliosis and glioma) and was considered pathogenic based on in silico analysis [41]. We checked its frequency in population databases, which was found to be incompatible with the disease prevalence (0.000279 and 0.00022 in TOPMed and gnomAD, respectively). Since this variant was initially identified in our cohort by classical screening through High Resolution Melting (HRM) analysis, the case was re-examined by NGS that identified a heterozygous frameshift variant c.7682_7683delAG in *NF1*, which is reported in ClinVar as pathogenic (class P according to ACMG criteria). In agreement with population data, the expression of the mutant minigene carrying the c.1062 + 113A > G variant does not affect splicing. Altogether, these findings exclude the pathogenicity of this intronic substitution. 

Among controls, we included two exonic variants. The synonym variant c.5694G > A p.(Glu1898=) was predicted to affect splicing by both the software tools we employed, although CADD score was below 15 and thus was considered not significant; in addition, allele frequency was 0.00014 (gnomAD). Notably, this synonym change was found in a patient who harbors the heterozygous canonical splicing variant c.288 + 1delG. In addition, the exonic substitution c.6882C > G p.(Leu2315=) was reported in ClinVar as likely benign and Mutation Taster predicted a splicing alteration. However, population data were against its pathogenic role (allele frequency of 0.000024 in TOPMed). In both cases, these synonym variants, when expressed from the minigene construct, did not affect transcript maturation in HEK293 cells. Again, population data and functional findings argue against a pathogenic role of these synonym changes.

### 2.2. Canonical and Non-Canonical NF1 Variants

The minigene-based assay was then employed to examine a series of 19 variants (14 intronic, 5 exonic) identified in patients with NF1 (Table 3).

**Figure 2:**
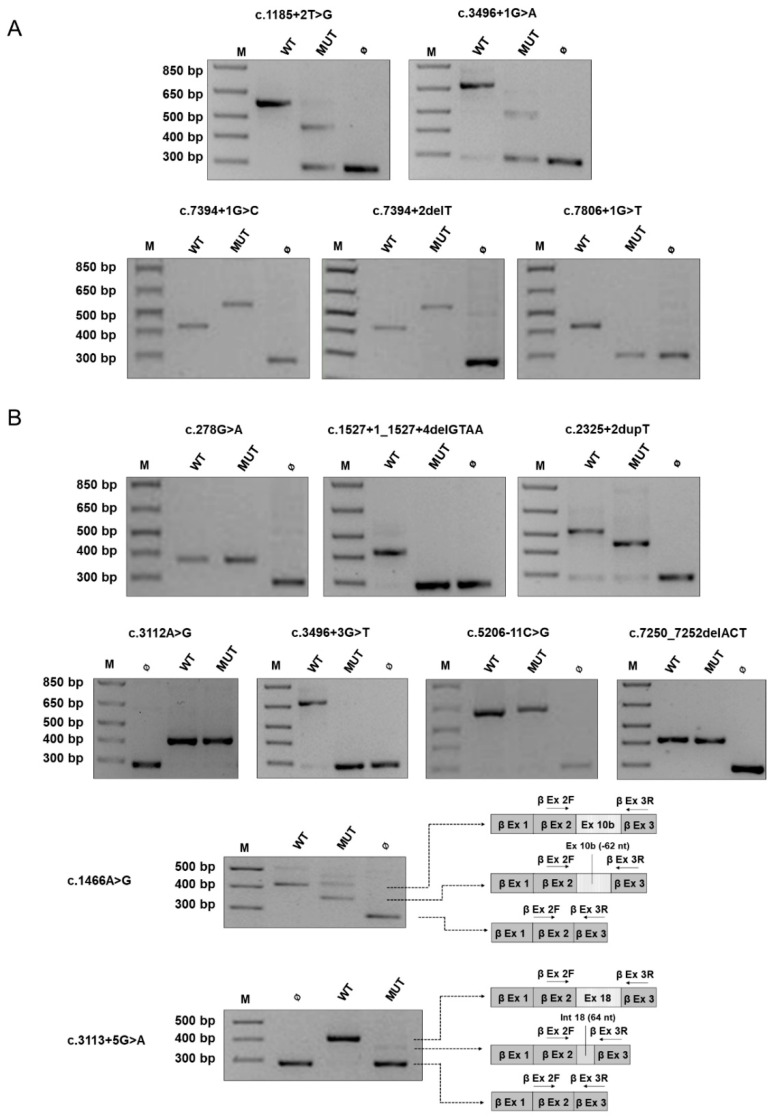
Canonical and non-canonical *NF1* variants. Agarose gel electrophoresis of RT-PCR products obtained after transfection of HEK293 cells with minigene constructs harboring *NF1* variants involving 5′ or 3′ GT/AG canonical splice sites (c.1185 + 2T > G, c.3496 + 1G > A, c.7394 + 1G > C, c.7394 + 2delT, c.7806 + 1G > T) (**A**) or other nucleotides outside the canonical splice sites (c.278G > A, c.1466A > G, c.1527 + 1_1527 + 4delGTAA, c.2325 + 2dupT, c.3112A > G, c.3113 + 5G > A, c.3496 + 3G > T, c.5206-11C > G, c.7250_7252delACT) (**B**). Cells were transfected with constructs harboring the indicated variants (MUT) or with the corresponding wild-type ones (WT), while the empty vector (Ø) was used as internal control where indicated. M, 1 Kb Plus DNA Ladder (ThermoFisher Scientific). Since the expression of the minigenes harboring the c.1466A > G and c.3113 + 5G > A variants yielded a splicing pattern characterized by more products, bands were Sanger sequenced and the results are schematically depicted next to gel images.

Concerning the 5 intronic substitutions affecting one of the two nucleotides at the canonical splice sites, all were predicted pathogenic by in silico tools. In all cases, the presence of the variant was associated with an aberrant splicing of the corresponding minigene expressed in HEK293 cells, with the absence of the wild-type transcript (Figure 2A). Skipping of the neighbor exon, which was observed in 3 variants, was the most common effect, whereas two intronic changes provoked intronic retention. These data confirm the pathogenicity of all these changes.

**Figure 3:**
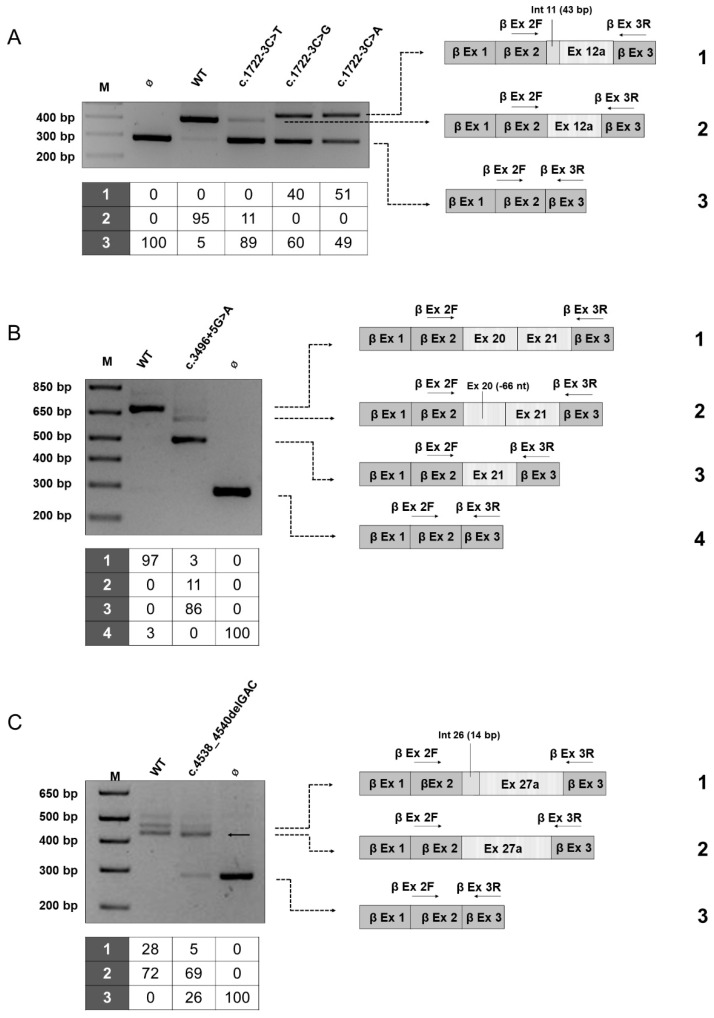
*NF1* variants associated with the residual production of wild-type transcript. On the left, agarose gel electrophoresis of RT-PCR products obtained after transfection of HEK293 cells transfected with the minigene constructs harboring the c.1722-3C > T, c.1722-3C > G, c.1722-3C > A (**A**), c.3496 + 5G > A (**B**), c.4538_4540delGAC (**C**) variants or with the corresponding wild-type ones (WT) or the empty vector (Ø). M, 1 Kb Plus DNA Ladder (ThermoFisher Scientific, Watham, MA, USA). On the right, a schematic representation of bands that were excised from each gel and Sanger sequenced (indicated with numbers from 1 to 4). The same gel bands were also quantified by densitometric analysis (performed with ImageJ) and the results are reported in the table below each gel image.

Among the other 14 variants outside the canonical splice sites (Figure 2B and Figure 3), expression of the minigene yielded a splicing pattern characterized by more products (Table 3) in seven cases, indicating the coexistence of multiple mechanisms altering transcript maturation, as for the c.3113 + 5G > A transition, which yielded two distinct transcripts when expressed in HEK293 cells: skipping of exon 18 (exon 23) and intronic retention of 64 nucleotides. In four other non-canonical variants, minigene assay revealed a single splicing aberrant product with exon skipping in three cases (c.1527 + 1_1527 + 4delGTAA, c.2325 + 2dupT, c.3496 + 3G > T), whereas the c.5206-11C > G substitution was associated with partial intronic retention, as shown by direct sequencing of the RT-PCR product (Supplementary Figure S1). 

For the c.3496 + 3G > T substitution, the minigene analysis showed skipping of exons 20 (exon 26) and 21 (exon 27), unlike the adjacent canonical splice site change c.3496 + 1G > A, which instead causes skipping of the neighboring exon 20 alone or of both exons 20 and 21, as shown by minigene assay; notably, cDNA analysis in a patient harboring this mutation detected only one aberrant transcript devoid of exon 20 [33].

The c.1466A > G p.(Tyr489Cys) is a recurrent *NF1* pathogenic variants, being reported 41 times in LOVD and in three unrelated patients of our cohort. This is a recognized example of missense change affecting transcript maturation, as shown by cDNA analysis of *NF1* [34] with loss of the last 62 nucleotides of exon 10b (exon 13) downstream to the substitution for the creation of a novel donor site. When expressed in the minigene system that examines single alleles, sequencing of RT-products revealed the presence also of the correctly spliced transcript (which harbors the r.1466a > g change, resulting in the amino acid substitution p.Tyr489Cys) that reaches significant levels of total products. These findings suggest that the c.1466 > G substitution acts with a double mechanism (at RNA and protein levels), causing both aberrant splicing and the substitution of the residue Tyr489, which is conserved in vertebrates (Supplementary Figure S2), underlining how distinct molecular mechanisms coexist to determine the pathogenicity of a nucleotide change. 

Interestingly, among the five exonic changes, three did not affect splicing at the minigene assay (Figure 2B). The c.278G > A p.(Cys93Tyr) missense substitution, which was predicted to marginally increase the strength of the donor site by MutationTaster, and was associated with a normal splicing pattern when expressed in HEK293 cells compared to the wild-type construct. This variant is rare (identified in a single individual in TOPMed, with allele frequency of 0.000008, absent in gnomAD), it was previously described in mutation screening analyses by protein truncation test (PTT)/heteroduplex test [47] and cDNA study [46] in NF1 patients and, in agreement with these data, our minigene assay confirms that this is a bona fide missense variant. This single nucleotide change was identified in a sporadic patient in our cohort, but DNA from family members was not available for segregation analysis. The Cysteine in position 93 is highly conserved throughout evolution (Supplementary Figure S2) and was found to be affected by two other distinct amino acid substitutions (Arg and Trp) in patients with NF1 [52,53], supporting the pathogenic role of missense variants affecting this codon (LP variant according to ACMG). Likewise, the c.3112A > G p.(Arg1038Gly) substitution, which is predicted to alter splicing by in silico analysis, does not affect transcript maturation at the minigene assay, thus behaving as a real missense change. This variant was previously found in seven NF1 patients from two unrelated families showing a specific phenotype without neurofibromas [23]. Although predicted to affect transcript maturation by Human Splicing Finder (HSF), the c.7250_7252delACT *inframe* deletion does not alter splicing pattern compared to the wild type when expressed from the *beta*-globin minigene, indicating that mRNA is correctly processed. However, this trinucleotide deletion, which is not reported in population databases nor in LOVD, HGMD, and ClinVar, determines the loss of a highly conserved tyrosine residue (Supplementary Figure S2) and is predicted pathogenic by in silico tools (Table 3). Furthermore, this variant was found in a sporadic case of NF1 and segregation analysis, confirming its de novo origin. All these data support a pathogenic role of this deletion, allowing to classify it as likely pathogenic (LP variant according to ACMG).

### 2.3. NF1 Variants Associated with the Residual Production of Wild-Type Transcript

The minigene assay unveiled three *NF1* variants causing aberrant splicing with concomitant residual production of wild-type transcript (Figure 3). 

**Figure 4:**
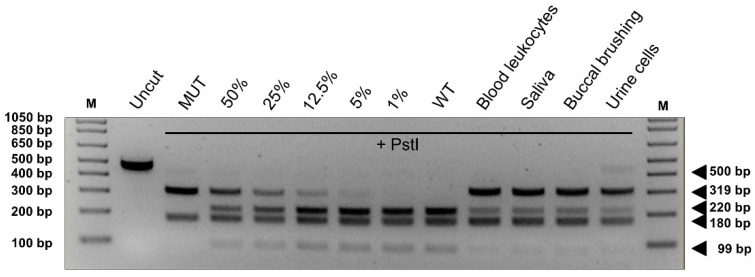
PCR-restriction fragment length polymorphism (PCR-RFLP) analysis by *PstI* digestion of the 500 bp long fragment of *NF1* exon 12a (exon 16) amplified from different samples (blood leukocytes, saliva, buccal brushing, and urine cells). Digestion of PCR products amplified from the wild-type (WT) or mutant (MUT) minigene constructs were included as control and mixed to create different mosaicism percentages (indicated above gel lanes and referring to the amount of mutant DNA molecules in the mix). M means molecular marker. The size of digested fragments is reported next to the gel image (uncut refers to not digested PCR).

We analyzed the effects on splicing of the de novo substitution c.1722-3C > T affecting the acceptor site of intron 11 (intron 15) that was found in an adult patient showing a specific phenotype characterized by cutaneous signs (CAL, SF), learning disabilities, and scoliosis without neurofibromas (Supplementary Table S1). This change, which was not previously described in NF1 patients, was reported in a single individual in the gnomAD Exomes database, showing an allele frequency of 0.000004. A different nucleotide change inserting a guanosine at the same residue was previously reported [15,35] and the cDNA analysis confirmed the generation of an aberrant transcript because of intronic retention generating a truncating product. Therefore, we employed the minigene assay to test the effects of all the three possible nucleotide changes in the position c.1722-3 at the acceptor site. As shown in Figure 3A, the minigene assay confirmed that the substitution with a purine (C > A or C > G) causes aberrant splicing with skipping of exon 12a (exon 16) and production of an out of frame transcript retaining 43 nucleotides of intron 11 (intron 15), thus abolishing the production of the wild-type mRNA. Conversely, the expression of the mutant construct harboring the c.1722-3C > T variant yielded two products: an aberrant transcript showing exon skipping along with the wild-type mRNA, which represents approximately 11% of the total transcripts as shown by densitometric analysis, indicating a hypomorphic effect on splicing. To exclude the possibility of a mosaicism, we examined the presence of the variant in DNA extracted from patient’s different tissues (blood leukocytes, saliva, buccal brushing, urine cells) by PCR-restriction fragment length polymorphism (RFLP) analysis, exploiting the abolition of a naturally occurring *PstI* restriction site by the 1722-3C > T change. As shown in Figure 4, the restriction pattern was compatible with a heterozygous status of the variation and was similar in all tissues, supporting the constitutive nature of the variant.

Another *de novo* substitution affecting the acceptor site outside the canonical dinucleotides, the c.3496 + 5 variant, allowed the production of residual wild-type mRNA (approximately 3% of the total) (Figure 3B). The remaining transcripts showed skipping of exon 20 (exon 26) (86%) or the inframe loss of the last 66 nucleotides of exon 20 (11%). This last aberrant mRNA derives from the creation of a novel acceptor site and results in the loss of 22 amino acids of exon 20 that belong to a highly conserved portion of neurofibromin (Supplementary Figure S2). Notably, while previous standard cDNA analysis performed by our group (Supplementary Figure S3) identified exon 20 skipping, the presence of the wild-type transcript or the product with intronic retention was not detected. Our minigene system allowed to unveil multiple mechanisms of missplicing, emphasizing the difficulties in predicting the functional consequences in these cases. Interestingly, this variant was detected in a young adult patient who has manifested only cutaneous signs so far (CALs and SF) (Supplementary Table S1).

Finally, the c.4538_4540delGAC deletion, which was identified in a sporadic patient with NF1, affected splicing at the minigene assay, with partial skipping of exon 27a (exon 34) but with the concomitant production of the correctly spliced transcript (that reaches 69% of total mRNA, a level very close to the wild-type control). This variant was absent in population and mutational databases and was predicted to affect splicing through the activation of a cryptic acceptor site. The deletion involves the highly conserved arginine 1513, which is part of the GTPase-activating protein (GAP)-related domain (GRD) of neurofibromin and accordingly showed a CADD score of 22.5 (with a pathogenic computational verdict that considers also the consequences at protein level). Segregation analysis revealed the presence of the variant in the mother, who did not manifest any clinical sign of the disorder, although mosaicism could not be ruled out. Altogether, these findings allowed to classify this deletion as a variant of unknown significance (class VUS ACMG).

## 3. Discussion

Hybrid minigenes represent a rapid and effective method to study the effects of genomic variations on splicing and have been extensively employed to functionally characterize variants in a number of genes, including *BRCA1* [54], *BRCA2* [55], *CFTR* [39,56], and *CHD7* [57]. This technique does not require patient’s RNA, overcoming the problem of analyzing transcripts with absent/low expression in leukocytes (as for *CFTR*) and avoiding the problem of illegitimate splicing that is observed in the case of long transcripts, such as *NF1*, if samples are not rapidly processed after venipuncture [28,29]. Actually, classical minigenes have another advantage: they can be used to analyze heterozygous mutations since, unlike classical cDNA analysis that usually does not permit to distinguish the transcripts derived from each allele, they study the maturation of messenger RNA generated from a single allele. This is particularly useful in the case of hypomorphic variants that allows the production of residual correctly spliced transcript, which would be otherwise masked by the mRNA generated by the wild-type allele with standard cDNA analysis. The ability of minigene assays to detect leakiness associated with novel variants is crucial since it may have phenotypic consequences [58].

All 10 *NF1* benign variants that we examined were found not to affect splicing at the minigene assays. It is of note that for this group of variations, in silico predictions would have not correctly computationally classified them as benign, underlining the importance of integrating bioinformatic data with experimental validation. 

As expected, all five *NF1* variants affecting one of the dinucleotides at canonical splice sites (c.1185 + 2T > G, c.3496 + 1G > A, c.7394 + 1G > C, c.7394 + 2delT, c.7806 + 1G > T) proved to be pathogenic by completely abolishing the correct splicing of the transcript. Likewise, for all nine intronic variants not involving the canonical splicing sites (c.1527 + 1_1527 + 4delGTAA, c.1722-3C > T, c.1722-3C > G, c.1722-3C > A, c.2325 + 2dupT, c.3113 + 5G > A, c.3496 + 3G > T, c.3496 + 5G > A, c.5206-11C > G), our in vitro minigene system allowed to confirm their pathogenicity, showing the production of aberrant transcripts. In particular, we observed that one of these variants, namely the c.5206-11C > G substitution, introduced a novel AG in the exclusion zone (AGEZ) between the authentic 3’ss AG and the branch point. The creation of a novel 3′ss causes intronic retention (type 3 splice effect) in accordance with previously published data [51].

Even coding region mutations independently on the effects of the corresponding amino acid can affect splicing if located in splice sites or within exonic splicing enhancers/silencers (ESE/ESS) regions or if generating cryptic GT-AG dinucleotides [59]. This is well known for *NF1*, where the disruption of functional ESE sequences by missense/nonsense changes is probably the most frequent mechanism underlying mutation-associated exon skipping [36]. In our series, we included seven exonic variants: two synonym substitutions that, as expected, were shown not to affect splicing and 5 missense variants/inframe deletions. Except for the c.1466A > G change, for all the other exonic variants, no splicing alterations were observed with the minigene assay: in three cases (c.278G > A, c.3112A > G, c.7250_7252delACT) population data, segregation analysis, previous findings [23,48,49] and in silico tools allowed to classify them as LP or P. In accordance with RNA studies performed by our group and others [35], these findings indicate that the c.1466A > G p.(Cys489Tyr) acts through two distinct mechanisms: in addition to the deleterious effect on splicing, a significant proportion of residual mutant protein is still produced, whose activity should be investigated by further functional studies. 

In the case of the c.4538_4540delGAC inframe deletion, minigene analysis identified the presence of multiple transcripts: beyond 26% of mRNA showing skipping of the inframe exon 27a (exon 34), cells transfected with the mutant minigene showed 69% of the correctly spliced product and 5% of mRNA retaining 14 nucleotides of intron 26 (intron 33). It should be noted that the product with intronic retention was expressed also by control cells transfected with the wild-type minigene, probably for the loss of intronic regulatory sequences within the construct. Nevertheless, in both cases, the levels of correctly spliced transcript were comparable, thus excluding a deleterious effect of the variation on splicing. The c.4538_4540delGAC variant was identified in a patient affected by sporadic NF1 and segregation analysis showed that the unaffected mother harbors the inframe deletion, although a mosaic state (that could explain her absence of symptoms [34]) could not be ruled out. This deletion is predicted to provoke the loss of the highly conserved arginine 1513, which lies within the GTPase-activating protein (GAP)-related domain (GRD) of neurofibromin. Functional data will be required to determine the consequences of this deletion at the protein level that now remains a VUS.

Our minigene assay proved to be useful in defining the molecular mechanism(s) of a variant, showing whether it acts as a bona fide missense variant/inframe deletion. This is a relevant point since it is the basis on which to design specific therapies tailored on the mutation-specific pathogenetic mechanism. 

The c.1722-3C > T variant at the acceptor splice site of intron 11 (intron 15), which has not been reported in patients with NF1 so far, was identified as de novo in an adult patient with sporadic disease. A distinct substitution C > G was previously described in *NF1* and cDNA analysis had shown a retention of 43 nucleotides of intron 11 [15,35]. We therefore employed minigene to examine the effects of all the possible nucleotide substitutions at this position of the acceptor site. Notably, the expression of the minigene harboring the C > G as well as the C > A substitution in HEK293 cells yielded two products: one retaining 43 nucleotides of intron 11 (as evident by cDNA analysis) and the other showing skipping of exon 12a (exon 16), indicating that the presence of a purine in -3 induces a severe effect on splicing. Conversely, the consequences of the C > T change on transcript maturation were less detrimental, leading to skipping of exon 12a, which was paralleled by the presence of approximately 11% levels of wild type product. 

Three distinct substitutions at the 5′ splice site (+1, +3 and +5) of intron 20 (intron 26) were investigated. The canonical c.3496 + 1G > A change was associated with skipping of exon 20 (exon 26) alone or of exons 20 and 21 (exon 27), whereas the c.3496 + 3G > T variant caused skipping not only of the adjacent exon, but also of exon 21. The occurrence of an abnormal skipping of two exons can be explained with a different order of intron removal in this region: the intron between the two exons is rapidly skipped compared to the two adjacent introns so that a big exon-like structure can be skipped [60]. As previously reported by Hori T and co-authors [61], the variant could change the order of intron skipping and cause a double exon skipping. Finally, also the c.3496 + 5G > A variant when expressed in the *beta*-globin minigene determined exon 20 (exon 26) skipping, which however coexists with an aberrant product (loosing 66 nucleotides of exon 20) and the correctly spliced transcript. These findings emphasize the importance of the sequence context and how adjacent changes in splice site regions or distinct variations at the same position might yield distinct effects on splicing maturation [62]. 

Interestingly, the c.1722-3C > T variant associated with leaky splicing and residual production of wild-type transcript, was confirmed to be de novo in a sporadic patient with NF1. It was previously shown that the levels of isoform-specific *NF1* mRNA correlates with the severity of the disease [63] and thus it is possible to speculate that residual levels of wild-type protein, at least in some tissues, may avoid the occurrence of some manifestations. A mild NF1 phenotype in a patient with a leaky *NF1* splicing mutation was previously reported, but it was combined with a complex mosaicism [64]. In the case of the c.1722-3C > T leaky splicing variant, the confounding effect of a possible mosaicism in the patient was excluded by PCR-RFLP analysis on DNA from distinct tissues. Although further studies are required to confirm this hypothesis, the possibility of leaky splicing in *NF1* underlining specific phenotypes should be considered and a rapid analysis with hybrid minigenes might be offered. Nevertheless, it remains to be determined which is the minimal level of full-length transcript required for normal function in different tissues.

Data from RNA classical analysis performed in our lab and by other groups [23,34,35,45,46,48] were available for 10 out of the 29 *NF1* variants of our series (Table 2 and Table 3). Remarkably, in four cases minigene assay revealed a more complex splicing profile compared to cDNA analysis, owing to the presence of multiple aberrant transcripts. Among the remaining six variant showing concordant results with the two strategies, three of them did not show any splicing alteration. Once more, these findings emphasize the usefulness of minigenes to characterize splicing profiles associated with *NF1* variants. 

The use of the minigene system may present some limitations, being an in vitro assay that requires the construction of an artificial hybrid gene and that could not detect tissue-specific missplicing. However, the correct processing of the wild-type construct usually ensures that the minigene respects all the regulatory elements required by the spliceosomal machinery to guarantee correct transcript maturation, and thus alterations observed with the mutant constructs are likely due to the presence of the mutation. In our series, some missplicing with exon skipping or intronic retention was detected also when expressing the wild type minigene, probably because the intronic portion included in the hybrid construct may lose some regulatory sequences. However, despite these limitations, in most cases the effect(s) of the single mutant allele on transcript maturation could be defined by comparing splicing patterns in cells transfected with the wild type or mutant construct. Of note, unlike all RNA-based methods, in the case of heterozygous variants this system is able to analyze transcripts generated by a single allele, thus avoiding the interference deriving from the wild-type gene. 

## 4. Materials and Methods 

### 4.1. Patients

We analyzed a series of 29 germinal variants in the *NF1* gene identified in patients referred to our center between 2005 and 2019 for a clinical diagnosis/suspicion of neurofibromatosis type 1 according to the NIH criteria [10,65]. Informed consent was obtained for each patient. Family history and relevant clinical data were analyzed from medical records. Table 1 reports the set of *NF1* variants that were examined in this work in familial or sporadic patients with NF1; their clinical features are described in Supplementary Table S1.

### 4.2. Molecular Analysis of the NF1 Gene

DNA was isolated from peripheral blood leucocytes using standard protocols. For RNA analysis, blood was collected in PAXgene whole blood samples (Becton Dickinson, Franklin Lakes, NJ, USA) and extraction was carried out with the PAXgene™ Blood RNA System (Qiagen, Hilden, Germany) according to the manufacturer’s instruction.

Over the years, different methods were employed for *NF1* molecular analysis that include: (i) mutation screening approaches (HRM analysis, as previously described [38]); (ii) cDNA analysis, which was performed by amplifying, after retrotranscription of 1 µg of total RNA, the whole coding region of *NF1* into five overlapping 2 kb amplicons [29,66] that were then directly sequenced through Sanger (Abi Prism 3700, ThermoFisher Scientific, Watham, MA, USA); (iii) finally, a NGS-based sequencing protocol consisting of library preparation with a Haloplex HS or SureSelect kits (Agilent Technologies, Santa Clara, CA, USA), which includes *NF1* and *SPRED1* genes, was employed and libraries were run on a MiniSeq apparatus (Illumina, San Diego, CA, USA).

Multiplex ligation-dependent probe amplification (MLPA) analysis was carried out for the screening of single/multiexon intragenic deletions/duplications using the SALSA MLPA kits P081/P082 (MRC Holland, Amsterdam, The Netherlands) according to manufacturer’s protocol.

When DNA was available from other family members, segregation analysis was carried out through bidirectional Sanger sequencing or MLPA analysis depending on the variation (Table 1). 

In the patient carrying the c.1722-3C > T variant, DNA was extracted also from other tissues (saliva, buccal brushing, and urine cells) using the Gentra Puregene kit (Puregene Cell and Tissue kit plus Qiagen GmbH, Hilden, Germany). The presence of the c.1722-3C > T variant in *NF1* intron 11 (intron 15) abolishes a naturally occurring *PstI* site and it was validated by a specific PCR-RFLP assay in different tissues: a 500 bp long fragment including exon 12a (exon 16) was amplified using specific primers and digested with *PstI* (New England Biolabs, Ipswich, MA, USA) one hour at 37 °C; restriction products were visualized on a 2.5% agarose gel.

### 4.3. Bioinformatic Analysis of Variants

Different complementary online software for splicing prediction were employed: Human Splicing Finder (HSF) Version 2.4.1 (http://www.umd.be/HSF/ Assessing on 21 December 2020), Mutation taster (http://www.mutationtaster.org/ Assessing on 21 December 2020), and Combined Annotation Dependent Depletion (CADD) v1.6 (https://cadd.gs.washington.edu/ Assessing on 21 December 2020). 

For mutation nomenclature, NM_000267.3 was used as reference sequence and *NF1* exons were numbered according to the historical numbering used by the NF1 community [26] (followed by the NCBI numbering in brackets).

Protein sequence alignments were performed using Multalin (http://multalin.toulouse.inra.fr/multalin/ Assessing on 28 December 2020).

The following population or disease databases were examined to check allele frequencies: the Genome Aggregation Database gnomAD (https://gnomad.broadinstitute.org/ Assessing on 28 December 2020), dbSNP (https://www.ncbi.nlm.nih.gov/snp/ Assessing on 28 December 2020), TOPMed (https://www.nhlbiwgs.org/ Assessing on 28 December 2020), Human Mutation Database (http://www.hgmd.cf.ac.uk Assessing on 28 December 2020), ClinVar (https://www.ncbi.nlm.nih.gov/clinvar/ Assessing on 28 December 2020), LOVD (https://www.lovd.nl/ Assessing on 28 December 2020). Variant interpretation was based on ACMG criteria and a detailed information on classification is reported in supplementary Table S2 [44].

### 4.4. Construction and Expression of the Minigenes

PCR fragments including the exon adjacent to each *NF1* variant and at least 100 bp of the upstream and downstream introns were amplified from patients’ genomic DNA and cloned into the *beta*-globin vector, as previously described [40]. In presence of short introns, a bigger portion of sequence including two adjacent exons was amplified and cloned in the minigene construct. One clone with the wild-type allele and one with the variant to test were retained for expression experiments and the correctness of each vector was verified by direct sequencing. HEK293 cells (6 × 10^5^) were transfected with 1 µg of the wild type or the mutated minigene using Lipofectamine 2000 (ThermoFisher Scientific, Watham, MA, USA) according to the manufacturer’s instruction. After 24 h total RNA was extracted with TRIzolTM (ThermoFisher Scientific, Watham, MA, USA), retrotranscribed using SuperScript II reverse transcriptase (ThermoFisher Scientific, Watham, MA, USA). The resulting cDNA was then amplified using specific primers for the *beta*-globin gene, designed on exon 2 (forward) and exon 3 (reverse), in order to avoid the amplification of eventual ectopically expressed *NF1* transcripts. PCR products were separated on a 2% agarose gel and individual bands were excised and sequenced using amplification primers (Sanger electropherograms of all *NF1* variants tested by minigene assay are reported in Supplementary Figure S1). When required, purified bands were subcloned using the TOPO TA Cloning kit (ThermoFisher Scientific, Watham, MA, USA) and single clones were sequenced as previously described [67]. A schematic representation of the minigene construct is depicted in Supplementary Figure S4.

Densitometric analyses were performed using the ImageJ (Image Processing and analysis in Java) software (https://imagej.nih.gov/ij/ Assessing on 28 December 2020).

## 5. Conclusions

With the fast entry of next-generation sequencing in the diagnostics of genetic diseases, the need for rapid, efficient, and reproducible systems to validate novel variants is warranted. This is relevant also for the (hopefully soon) clinical use of mutation-targeted therapy for NF1, as well as for other genetic disorders. Our minigene-based approach represents a valid tool to experimentally validate variants potentially causing missplicing, with the invaluable advantage of detecting leaky splicing that may underlines hypomorphic alleles.

## Figures and Tables

**Table 1:** *NF1* variants studied in this work and segregation data.

Variant [HGVS)	Familial/Sporadic Disease	Segregation Analysis
**Benign Variant**		
c.205-23G > A (c.974delT)	F (mother, brother, sister)	n.a.
c.289-75_289-74insTG	S ^1^	Variant inherited from the unaffected mother
c.1062 + 113A > G (c.7682_7683delAG)	S	n.a.
c.1393-82dupT ^2^	F (mother, sister, maternal aunt)	Variant identified in mother, sister, unaffected maternal aunt and grandfather)
c.4111-8_4111-6delGTT	S	Variant inherited from the unaffected father
c.5694G > A p.(Glu1898=) (288 + 1delG)	S	n.a.
c.6882C > G ^2^ p.(Leu2294=)	F (father, paternal grandmother and aunt)	n.a.
c.7259-17C > T (exon 39 deletion)	S	n.a.
c.8051-30G > A	S	Variant inherited from the unaffected mother
c.8097 + 55T > C (exon 14 deletion)	S	n.a.
**Canonical Variant**		
c.1185 + 2T > G	U	n.a.
c.3496 + 1G > A	S	De novo
c.7394 + 1G > C	S	De novo
c.7394 + 2delT	S	De novo
c.7806 + 1G > T	S	De novo
**Non-canonical Variant**		
c.278G > A p.(Cys93Tyr)	F (mother, brother, son)	Variant identified in affected son (mother and brother not tested)
c.1466A > G ^3^ p.(Tyr489Cys)	S	De novo
c.1527 + 1delGTAA	F (mother, brother, maternal uncle and grandmother)	Variant identified in affected mother and brother (other family member not tested)
c.1722-3C > T	S	De novo
c.1722-3C > G ^4^	-	-
c.1722-3C > A ^4^	-	-
c.2325 + 2dupT	U	n.a.
c.3112A > G ^5^ p.(Arg1038Gly)	Case 1: F (mother, maternal uncle and 1st cousin; son, daughters); Case 2: S	Case 1: Variant identified in affected mother, 1st maternal cousin, son, daughters (maternal uncle not tested); Case 2: De novo
c.3113 + 5G > A	U	n.a.
c.3496 + 3G > T	U	n.a.
c.3496 + 5G > A	S	De novo
c.4538_4540delGAC p.(Arg1513del)	S	Variant inherited from the unaffected mother
c.5206-11C > G	F (mother)	n.a.
c.7250_7252delACT p.(Tyr2417del)	S	De novo

For the benign variations included in the study, when available, the pathogenic variant in *NF1* identified in the same patient is indicated into brackets. ^1^ Mosaic NF1 (Lisch nodules in the left eye, 4 café-au-lait spots (CALs)). ^2^ Several family members present multiple CALs, without other signs of NF1. ^3^ This substitution was identified in three unrelated sporadic patients of our NF1 cohort (in all the cases segregation analyses demonstrated the de novo origin of the variant). ^4^ These two variants were not identified in our cohort but were used as controls in the minigene assay (the c.1722-3C > G was previously described [15,35]). ^5^ This variant was previously reported in patients from two unrelated families [23]. Abbreviations: Familial (F) or sporadic (S) or unknown (U) refers to the disease. Segregation analysis indicates whether the *NF1* variant was *de novo* or identified in other family members; n.a. means not available (no family members were available for analysis).

**Table 2:** Bioinformatics analysis and minigene splicing outcomes of benign *NF1* variants.

Genomic Coordinate	cDNA Change	Effect on RNA ^1^	Effect on Protein	Location	GnomAD Allele Frequency ^2^	TOPMED Allele Frequency	Human Splicing Finder	Mutation Taster	CADD Score	LOVD, HGMD, ClinVar ^3^	References	Splicing Alterations on Minigene	ACMG Class ^3^
29486005-G-A	c.205-23G > A			intron 2 (intron 2)	1/249602 (0.000004006) (E)	–	No significant impact on splicing signals.	Acceptor site marginally increased	14.08	- - -	–	No	B
29490130--TG	c.289-75_289-74insTG	No ^1^		intron 3 (intron 3)	–	–	No significant impact on splicing signals.	Acceptor site marginally increased; acceptor site increased.	12.91	- - -	–	No	B
29527726-A-G	c.1062 + 113A > G			intron 7 (intron 9)	7/31406 (0.00022) (G)	35/125568 (0.000279)	New donor splice site: Activation of a cryptic donor site. Potential alteration of splicing.	No significant impact on splicing signals.	0.315	P CS1512923 -	[41]	No	B
29541383--T	c.1393-82dupT			intron 10a (intron 12)	2/31276 (0.00006) (G)	9/125568 (0.000072)	No significant impact on splicing signals.	No significant impact on splicing signals.	3.171	- - -	–	No	B
29585354-GTT-	c.4111-8_4111-6delGTT			intron 23a (intron 30)	116/251202 (0.00046) (E)	67/125568 (0.000534)	No significant impact on splicing signals.	Acceptor site increased; acceptor site marginally decreased.	14.57	B - LB	[42]	No	B
29657461-G-A	c.5694G > A		p.(Glu1898=)	exon 30 (exon 38)	35/251248 (0.000139) (E)	9/125568 (0.000072)	Alteration of auxiliary sequences: Significant alteration of exonic splicing enhancers/silencers (ESE/ESS) motifs ratio.	Donor site marginally increased; donor site gained.	11.45	B - LB/VUS	–	No	B
29667546-C-G	c.6882C > G		p.(Leu2294=)	exon 38 (exon 46)	1/251486 (0.000004) (E)	3/125568 (0.000024)	No significant impact on splicing signals.	Acceptor site marginally increased; donor site gained.	10.03	- - LB	–	No	B
29677184-C-T	c.7259-17C > T			intron 40 (intron 48)	603/251342 (0.002399) (E)	940/125568 (0.007486)	No significant impact on splicing signals.	No significant impact on splicing signals.	3.685	B CS00088 B	[43]	No	B
29685957-G-A	c.8051-30G > A			intron 46 (intron 54)	37/250938 (0.0001474) (E)	22/125568 (0.000175)	Alteration of auxiliary sequences: Significant alteration of ESE/ESS motifs ratio	No significant impact on splicing signals.	3.438	LB - -	–	No	B
29686088-T-C	c.8097 + 55T > C			intron 47 (intron 55)	16/31406 (0.00051) (G)	66/125568 (0.000526)	Alteration of auxiliary sequences: Significant alteration of ESE/ESS motifs ratio.	No significant impact on splicing signals.	3.611	- - -	–	No	B

^1^ RNA analysis performed in our laboratory; ^2^ E, gnomAD exomes; G, gnomAD genomes; ^3^ P, pathogenic; LP, likely pathogenic; VUS, variant of uncertain significance; LB; likely benign; B, benign [44].

**Table 3:** Bioinformatics analysis and minigene splicing outcomes of *NF1* variants affecting canonic splice sites (above) or other sites (below).

Genomic Coordinate	cDNA Change	Effect on RNA ^1^	Effect on Protein	Location	GnomAD Allele Frequency ^2^	TOPMED Allele Frequency	Human Splicing Finder	Mutation Taster	CADD Score	LOVD, HGMD, ClinVar ^3^	References	Splicing Alterations on Minigene	ACMG Class ^3^
**Canonical variants**													
29528179-T-G	c.1185 + 2T > G			intron 8 (intron 10)	–	–	Alteration of the WT donor site, most probably affecting splicing.	Alteration within used splice site, likely to disturb normal splicing; donor site lost, donor site marginally increased, donor site gained.	34	P - P	–	Skipping of exon 8 (10) alone and of both exon 7 (9) and 8 (10)	P
29559900-G-A	c.3496 + 1G > A	Skipping of exon 20: r.3315_3496del182 [34]		intron 20 (intron 26)	–	–	Alteration of the WT donor site, most probably affecting splicing.	Alteration within used splice site, likely to disturb normal splicing; donor site lost; acceptor site marginally increased; donor site increased; donor gained.	33	- CS072245 -	[34]	Skipping of exon 20 (26) alone and of both exon 20 (26) and 21 (27)	P
29677337-G-C	c.7394 + 1G > C			intron 41 (intron 49)	–	–	Alteration of the WT donor site, most probably affecting splicing.	Alteration within used splice site, likely to disturb normal splicing; donor site lost; acceptor site increased; donor marginally increased.	35	- - -	–	Intronic retention of 126 nucleotides	P
29677338-T-	c.7394 + 2delT			intron 41 (intron 49)	–	–	Alteration of the WT donor site, most probably affecting splicing	Alteration within used splice site, likely to disturb normal splicing; donor site lost; acceptor site marginally increased; acceptor site increased; donor site increased: acceptor site gained; donor site gained.	32	- - -	–	Intronic retention of 126 nucleotides	P
29684109-G-T	c.7806 + 1G > T	r.7676_7806del131 [35,45]	p.(Glu2559Glyfs*10)	intron 44 (intron 52)	–	–	Alteration of the WT donor site, most probably affecting splicing	Alteration within used splice site, likely to disturb normal splicing; donor site lost; acceptor site increased; acceptor site marginally increased; acceptor site gained.	34	P CS031796 -	[35,45]	Skipping of exon 44 (52)	P
**Non-canonical variants**													
29486101-G-A	c.278G > A	no effect on splicing: r.278g > a [46]	p.(Cys93Tyr)	exon 3 (exon 3)	–	1/125568 (0.000008)	No significant impact on splicing signals.	Donor site marginally increased.	29.5	LP CM001252 P/VUS	[46,47]	No	LP
29541542-A-G	c.1466A > G	r.(1466a > g, 1466_1527del62) ^1^ [35]	p.(Tyr489Cys, Tyr489*)	exon 10b (exon 13)	3/250646 (0.000012) (E)	–	Significant alteration of ESE/ESS motifs ratio; activation of a cryptic donor site. Potential alteration of splicing.	Donor site increased.	23.9	P CM1111787 P	[15,35,48,49,50]	Loss of 62 nucleotides of exon 10b (13) downstream the mutation; production of the correctly spliced transcript	P
29541604-GTAA-	c.1527 + 1_1527 + 4delGTAA	r.1393_1527del135 [35,45,50]	p.(Ser465_Cys509del)	intron 10b (intron 13)	–	–	Alteration of the WT donor site, most probably affecting splicing.	Alteration within used splice site, likely to disturb normal splicing; donor site lost, acceptor site gained.	33	- - LP	[35,45,50]	Skipping of exon 10b (13)	P
29550459-C-T	c.1722-3C > T			intron 11 (intron 15)	1/250978 (0.000004) (E)	–	Activation of a cryptic donor site. Potential alteration of splicing	Alteration within used splice site, likely to disturb normal splicing; acceptor site lost, acceptor site increased.	20.2	- - -	–	Skipping of exon 12a (16) and production of WT transcript	LP
29550459-C-G	c.1722-3C > G	r.1721_1722ins1722-43_1722-1 [35]	p.(Ser574Argfs*28)	intron 11 (intron 15)	–	–	Alteration of the WT acceptor site, most probably affecting splicing	Alteration within used splice site, likely to disturb normal splicing; acceptor site lost, donor site gained.	25.1	P CS000051 -	[15,35]	Skipping of exon 12a (16) and production of a transcript retaining 43 nucleotides of intron 11 (15)	LP
29550459-C-A	c.1722-3C > A			intron 11 (intron 15)	–	–	Alteration of the WT acceptor site, most probably affecting splicing	Alteration within used splice site, likely to disturb normal splicing; acceptor site lost, donor site marginally increased, donor site gained.	24.1	- - P	–	Skipping of exon 12a (16) and production of a transcript retaining 43 nucleotides of intron 11 (15)	LP
29554312--T	c.2325 + 2dupT			intron 14 (intron 19)	–	–	Alteration of the WT donor site, most probably affecting splicing; activation of a cryptic donor site. Potential alteration of splicing.	Donor site lost; donor site gained.	23	- - -	–	Skipping of exon 14 (19)	P
29557399-A-G	c.3112A > G	r.3112a > g [23]	p.(Arg1038Gly)	exon 18 (exon 23)	–	–	Significant alteration of ESE/ESS motifs ratio.	Donor site lost; donor site marginally increased.	29.1	LP - -	[23]	No	P
29557405-G-A	c.3113 + 5G > A	r.2991_3113del123 [34,35]	p.(Tyr998_Arg1038del)	intron 18 (intron 23)	–	–	Alteration of the WT donor site, most probably affecting splicing.	Donor site decreased; donor site marginally increased; donor site increased.	23.1	- CS072240 P	[34,35]	Skipping of exon 18 (23) and production of a transcript devoid of exon 18 (23) retaining 64 nucleotides of intron 18 (23)	P
29559902-G-T	c.3496 + 3G > T			intron 20 (intron 26)	–	–	Alteration of the WT donor site, most probably affecting splicing.	Acceptor site marginally increased; donor site decreased; donor site increased.	21.7	- - -	–	Skipping of exon 20 (26) and 21 (27)	P
29559904-G-A	c.3496 + 5G > A	r.3315_3496del182 ^1^		intron 20 (intron 26)	–	–	Alteration of the WT donor site, most probably affecting splicing.	Acceptor site marginally increased; donor site increased; donor site lost; donor site increased; donor site marginally increased; donor site gained.	23.0	- - VUS	–	Skipping of exon 20 (26), loss of the last 66 nucleotides of exon 20 (26) and production of WT transcript.	P
29588752-GAC-	c.4538_4540delGAC		p.(Arg1513del)	exon 27a (exon 34)	–	–	Significant alteration of ESE/ESS motifs ratio; activation of a cryptic acceptor site. Potential alteration of splicing.	Donor site increased. Disease causing at protein level.	22.5	- - -	–	Skipping of exon 27a (34); production of correctly spliced transcript	VUS
29654506-C-G	c.5206-11C > G			intron 28 (intron 36)	–	–	Alteration of the WT acceptor site, most probably affecting splicing; activation of a cryptic acceptor site. Potential alteration of splicing.	Acceptor site marginally increased; acceptor site increased.	16.7	- - -	–	Intronic retention of 10 nucleotides ^4^	LP
29676261-ACT-	c.7250_7252delACT		p.(Tyr2417del)	exon 40 (intron 48)	–	–	Alteration of the WT donor site, most probably affecting splicing; activation of a cryptic donor site. Activation of a cryptic acceptor site. Potential alteration of splicing.	No significant impact on splicing signals. Disease causing at protein level.	19.9	- - -	–	No	LP

^1^ RNA analysis performed in our laboratory; ^2^ E, gnomAD exomes; G, gnomAD genomes; ^3^ P, pathogenic; LP, likely pathogenic; VUS, variant of uncertain significance; LB; likely benign; B, benign [44]; ^4^ AG creation in the AGEZ zone [51].

## Data Availability

The data presented in this study are available in the article and Supplementary Materials.

## Supplementary Materials

The following are available online at https://www.mdpi.com/2072-6694/13/5/999/s1, Figure S1: Sanger sequencing of the RT-PCR products obtained after transfection of HEK293 cells with the minigene construct harboring the indicated variant (MUT) or with the corresponding wild-type one (WT). Figure S2: Multalin alignments of wild-type and mutant (p.Cys93Tyr, p.Tyr489Cys, p.Arg1513del, p.Tyr2417del and p.Thr1145_Gly1166del) human neurofibromin with orthologues of different species. Figure S3: cDNA analysis of *NF1* in the patient harboring the c.3496 + 5G > A variant. Figure S4: A schematic representation of the hybrid minigene construct used in the experiments. Table S1: clinical features observed in families analyzed in this study. Table S2: ACMG classification of *NF1* variants studied in this work (based on criteria reported in [44]).
