# Supplementary material for: Hybrid Minigene Assay: An Efficient Tool to Characterize mRNA Splicing Profiles of NF1 Variants
Source: Cancers (Basel). 2021 Feb 27;13(5):999. doi: 10.3390/cancers13050999 (PMC7957615; doi:10.3390/cancers13050999)
Supplement: Supplementary file 1 [file cancers-13-00999-s001.zip › Supplementary tables.docx]

**Table S1: clinical features observed in families analyzed in this study**

| **Family** | ***NF1* Variant (NM_000267.3)** | **Phenotype ^1^** |
| --- | --- | --- |
| **Benign variants** |  |  |
| Family 1 proband, M, 58 yrs | c.205-23G>A (c.974delT) | CAL, SF, OS, >100 DNF |
| Family 2 proband, F, 31 yrs | c.289-75_289-74insTG | CAL, OS ^2^ |
| Family 3: proband, , 12 yrs | c.1062+113A>G (c.7682_7683delAG) | CAL, SF, Mc |
| Family 4 ^3^: proband, M, 13 yrs | c.1393-82dupT | CAL LD ^3^ |
| Family 4: mother, 43 yrs | c.1393-82dupT | CAL |
| Family 4: sister, F, 18 yrs | c.1393-82dupT | CAL |
| Family 4: maternal aunt, 37 yrs | c.1393-82dupT not detected | CAL |
| Family 4: maternal aunt, 45 yrs | c.1393-82dupT | unaffected |
| Family 5 proband, F, 8 yrs | c.4111-8_4111-6delGTT | CAL, SF |
| Family 5: father, 40 yrs | c.4111-8_4111-6delGTT | unaffected |
| Family 6: proband, M, 44 yrs | c.5694G>A p.(Glu1898=) (288+1delG) | CAL, SF, > 50 DNF, SNF |
| Family 7 ^3^: proband, M, 9 yrs | c.6882C>G p.(Leu2294=) | CAL, GHD |
| Family 7: father, 46 yrs | c.6882C>G p.(Leu2294=) | CAL, SS |
| Family 8: proband, M, 13 yrs | c.7259-17C>T (exon 39 deletion) | CAL, SF, OS, PNF |
| Family 9: proband, F, 11 yrs | c.8051-30G>A | CAL, SF, SC |
| Family 10: proband, F, 18 yrs | c.8097+55T>C (exon 14 deletion) | CAL, SF, LD, DNF, SC |
| **Canonical variants** |  |  |
| Family 11: proband, F, 41 yrs | c.1185+2T>G | Clinical diagnosis of NF1 confirmed in another center |
| Family 12. proband, M, 22 yrs | c.3496+1G>A | CAL, SF, OS, DNF, large PNF, LD |
| Family 13: proband, M, 11 yrs | c.7394+1G>C | CAL, SF |
| Family 14: proband, M, 22 yrs | c.7394+2delT | CAL, SF, MC, OS, OPG, PNF, DNF, LD |
| Family 15: proband, M, 8 yrs | c.7806+1G>T | CAL, SF, OS, LD, SC |
| **Non canonical variants** |  |  |
| Family 16: proband, F, 49 yrs | c.278G>A p.(Cys93Tyr) | CAL, SF, >100 DNF, OS |
| Family 16: son, 14 yrs | c.278G>A p.(Cys93Tyr) | CAL, MC |
| Family 17: proband, M, 10 yrs | c.1466A>G ^3^ p.(Tyr489Cys) | CAL, SF, OS; OPG, PNF |
| Family 18 proband, M, 53 yrs | c.1466A>G ^3^ p.(Tyr489Cys) | CAL, SF, OS, DNF, PNF, HBP, glaucoma, cavernous cerebellar angioma |
| Family 19 proband, M, 22 yrs | c.1466A>G ^3^ p.(Tyr489Cys) | CAL, SF, DNF, OS, LD |
| Family 20 proband, F, 20 yrs | c.1527+1delGTAA | CAL, SF, OS, DNF, PNF, SO, OPG, SWD, MC |
| Family 20 mother, 52 yrs | c.1527+1delGTAA | CAL, SF, OS, DNF, MC |
| Family 20 brother, M, 16 yrs | c.1527+1delGTAA | CAL, SF, OS, PNF, MC, SC, LD |
| Family 21 proband, F, 25 yrs | c.1722-3C>T | CAL, SF, SC, LD, SZ ^4^ |
| Family 22 proband, F, 26 yrs | c.2325+2dupT | CAL, SF, OS, SZ |
| Family 23 proband, F, 32 yrs | c.3112A>G | CAL, SF, SS, MC, FNF |
| Family 24 proband, F, 42 yrs | c.3112A>G ^5^ | CAL, SF, FNF |
| Family 25 proband, M, 11 yrs | c.3113+5G>A | Clinical diagnosis confirmed of NF1 in another center |
| Family 26 proband, M, 6 yrs | c.3496+3G>T | CAL, SF, LD |
| Family 27 proband, F, 18 yrs | c.3496+5G>A | CAL, SF |
| Family 28 ^3^: proband, M, 11 yrs | c.4538_4540delGAC | CAL, SS |
| Family 28: mother | c.4538_4540delGAC | unaffected |
| Family 29: proband, M, 19 yrs | c.5206-11C>G | CAL, SF, OS, LD, SS, SC |
| Family 29: mother, 52 yrs | Not tested | CAL, SF, OS, DNF, SS, SC |
| Family 30: proband, M, 14 yrs | c.7250_7252delACT | CAL, SF, OS, MC |

^1^ Abbreviations: CALs = café-au-lait spots; SF = skinfold freckling; OS = ocular signs (Irish nodules and / or choroidal abnormalities); OPG = optic pathway glioma; PNF = plexiform neurofibroma; DNF = dermal neurofibroma; SNF = spinal neurofibroma; LD = learning disabilities; GHD = growth hormone defect; SS = short stature; SC = scoliosis; MC = macrocephaly; HBP = high blood pressure; SWD = sphenoid wing dysplasia; SZ = seizures; FNF = Facial Noonan features n.a. not available

^2^ The patient manifested a mosaic NF1 due to segmental distribution of CAL and OS confined to the left eye.

^3^ These families do not fulfill the diagnostic criteria for NF1.

^4^ The patient manifested seizures during childhood consequent to a cortical dysplasia, which completely regressed after surgical resection of the brain lesion.

^5^ Clinical data of all the other family members have been described elsewhere [23].

**Table S2: ACMG classification of *NF1* variants studied in this work (based on criteria reported in [44].**

| **Genomic coordinate** | **c.DNA change** | **ACMG criteria** | **ACMG Class** |
| --- | --- | --- | --- |
| **Benign variants** |  |  |  |
| 29486005-G-A | c.205-23G>A | PM2, BS3, BP2, BP4 | LB |
| 29490130--TG | c.289-75_289-74insTG | PM2, PP3, BS3, BS4 | B |
| 29527726-A-G | c.1062+113A>G | BS2, BS3, BP2, BP4, | B |
| 29541383--T | c.1393-82dupT | BS1, BS3, BS4, BP4 | B |
| 29585354-GTT- | c.4111-8_4111-6delGTT | PP3,BS1, BS2, BS3, BS4 | B |
| 29657461-G-A | c.5694G>A | BS2, BS3, BP2, BP4, BP7 | B |
| 29667546-C-G | c.6882C>G | BS1, BS3, BP4, BP6, BP7 | B |
| 29677184-C-T | c.7259-17C>T | BS1, BS2, BS3, BP2, BP4, BP6 | B |
| 29685957-G-A | c.8051-30G>A | BS2, BS3, BS4, BP4 | B |
| 29686088-T-C | c.8097+55T>C | BS2, BS3, BP2, BP4 | B |
| **Canonical Variants** |  |  |  |
| 29528179-T-G | c.1185+2T>G | PVS1, PS3, PM2, PP3, PP5 | P |
| 29559900-G-A | c.3496+1G>A | PVS1, PS3, PM2, PM6, PP3 | P |
| 29677337-G-C | c.7394+1G>C | PVS1, PS3, PM2, PM6, PP3 | P |
| 29677338-T- | c.7394+2delT | PVS1, PS3, PM2, PM6, PP3 | P |
| 29684109-G-T | c.7806+1G>T | PVS1, PS3, PM2, PM6, PP3 | P |
| **Non-canonical variants** |  |  |  |
| 29486101-G-A | c.278G>A | PM1, PM2, PM5, PP1, PP2, PP3, PP5, BS3 | LP |
| 29541542-A-G | c.1466A>G | PS3, PM2, PM6, PP2, PP3, PP5 | P |
| 29541604-GTAA- | c.1527+1_1527+4delGTAA | PS3, PM2, PP1, PP3, PP5 | P |
| 29550459-C-T | c.1722-3C>T | PS3, PM2, PM6, PP3 | LP |
| 29550459-C-G | c.1722-3C>G | PS3, PM2, PP3, PP5 | LP |
| 29550459-C-A | c.1722-3C>A | PS3, PM2, PP3, PP5 | LP |
| 29554312--T | c.2325+2dupT | PS3, PM2, PP3 | LP |
| 29557399-A-G | c.3112A>G | PM2, PM6, PP1, PP2, PP3, BS3 | LP |
| 29557405-G-A | c.3113+5G>A | PS3, PM2, PP3, PP5 | LP |
| 29559902-G-T | c.3496+3G>T | PS3, PM2, PP3 | LP |
| 29559904-G-A | c.3496+5G>A | PS3, PM2, PM6, PP3 | LP |
| 29588752-GAC- | c.4538_4540delGAC | PM1, PM2, PM4, PP3, BS3, BS4 | VUS |
| 29654506-C-G | c.5206-11C>G | PS3, PM2, PP3 | LP |
| 29676261-ACT- | c.7250_7252delACT | PM2, PM4, PM6, PP3, BS3 | LP |
