# Supplementary material for: Hybrid Minigene Assay: An Efficient Tool to Characterize mRNA Splicing Profiles of NF1 Variants
Source: Cancers (Basel). 2021 Feb 27;13(5):999. doi: 10.3390/cancers13050999 (PMC7957615; doi:10.3390/cancers13050999)
Supplement: Supplementary file 1 [file cancers-13-00999-s001.zip › Supplementary Figures.pdf]

Figure S1

BENIGN VARIANTS

MUT (c.205-23G>A)

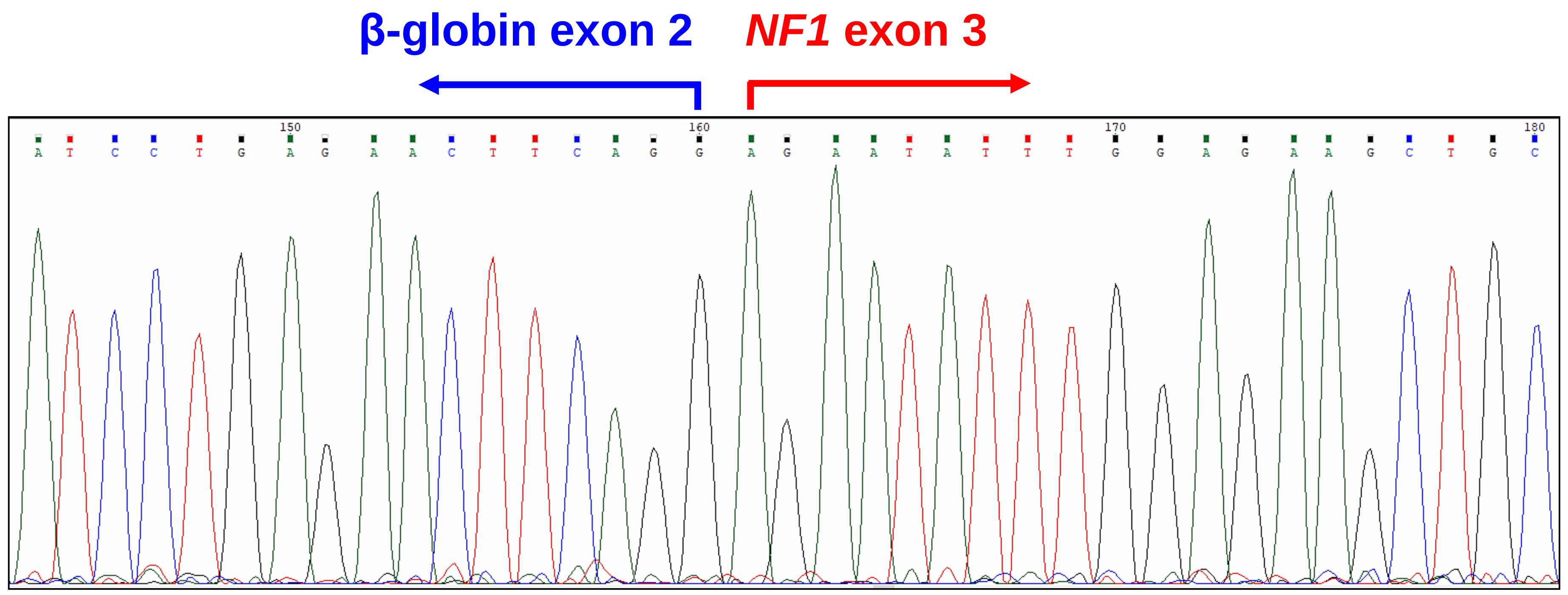

MUT (c.289-75_289-74insTG)

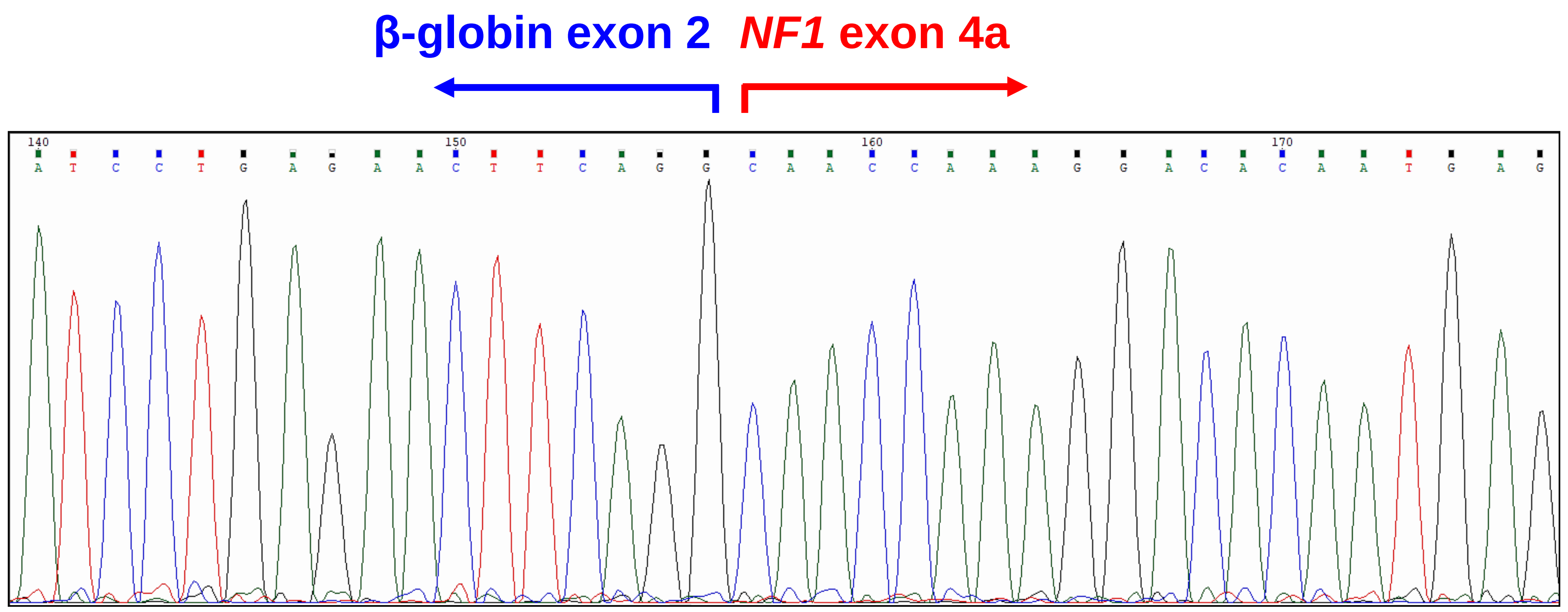

MUT (c.1062+113A>G)

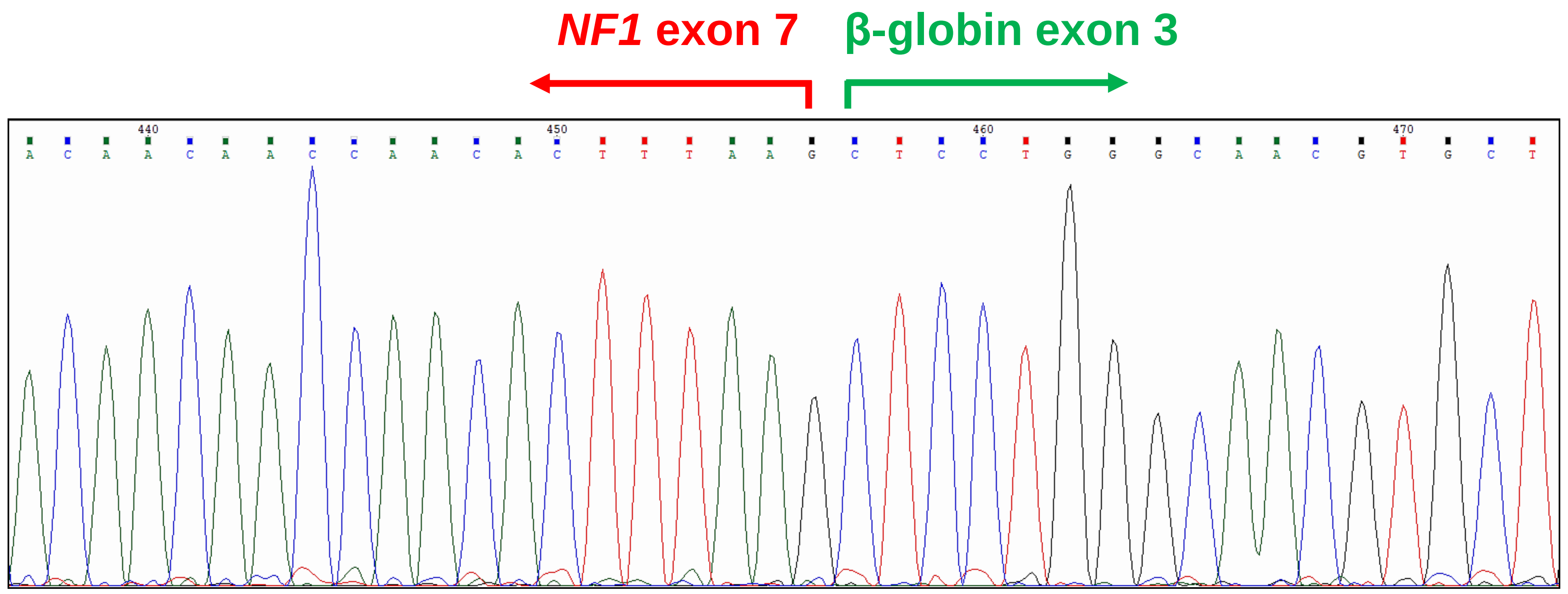

MUT (c.1393-82dupT)

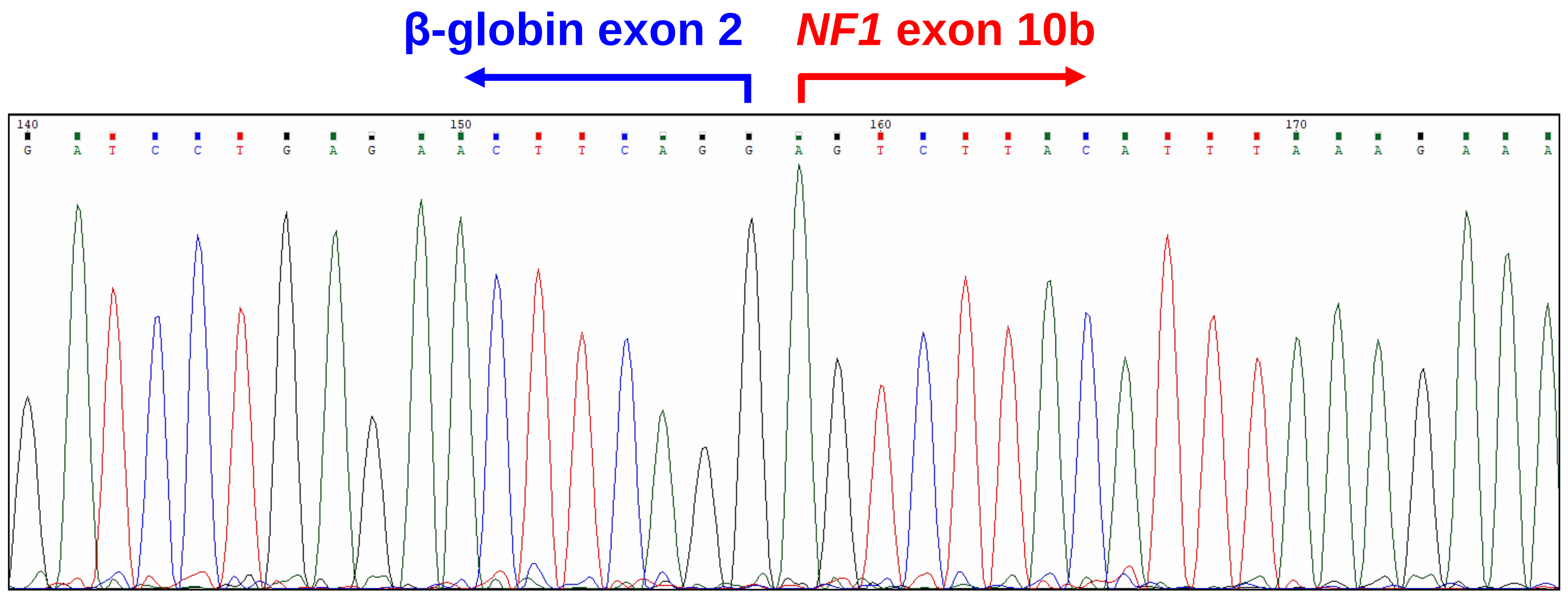

MUT (c.4111-8_4111-6delGTT)

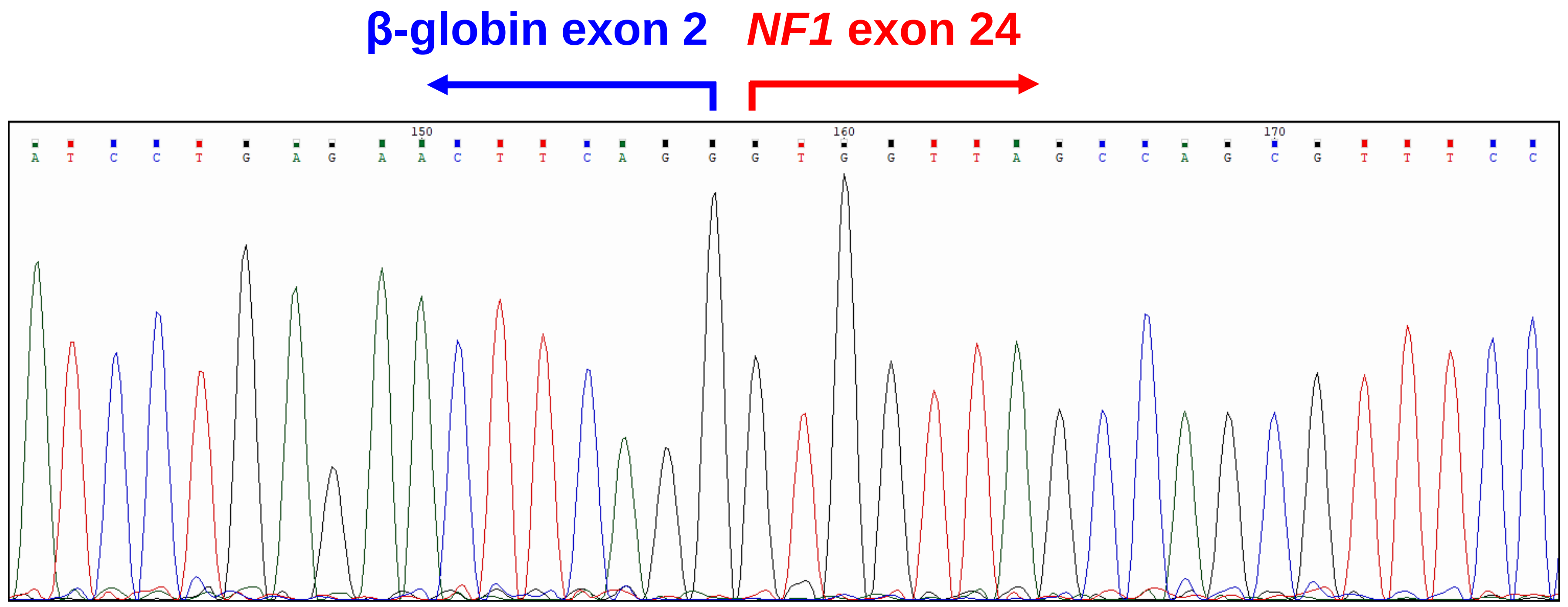

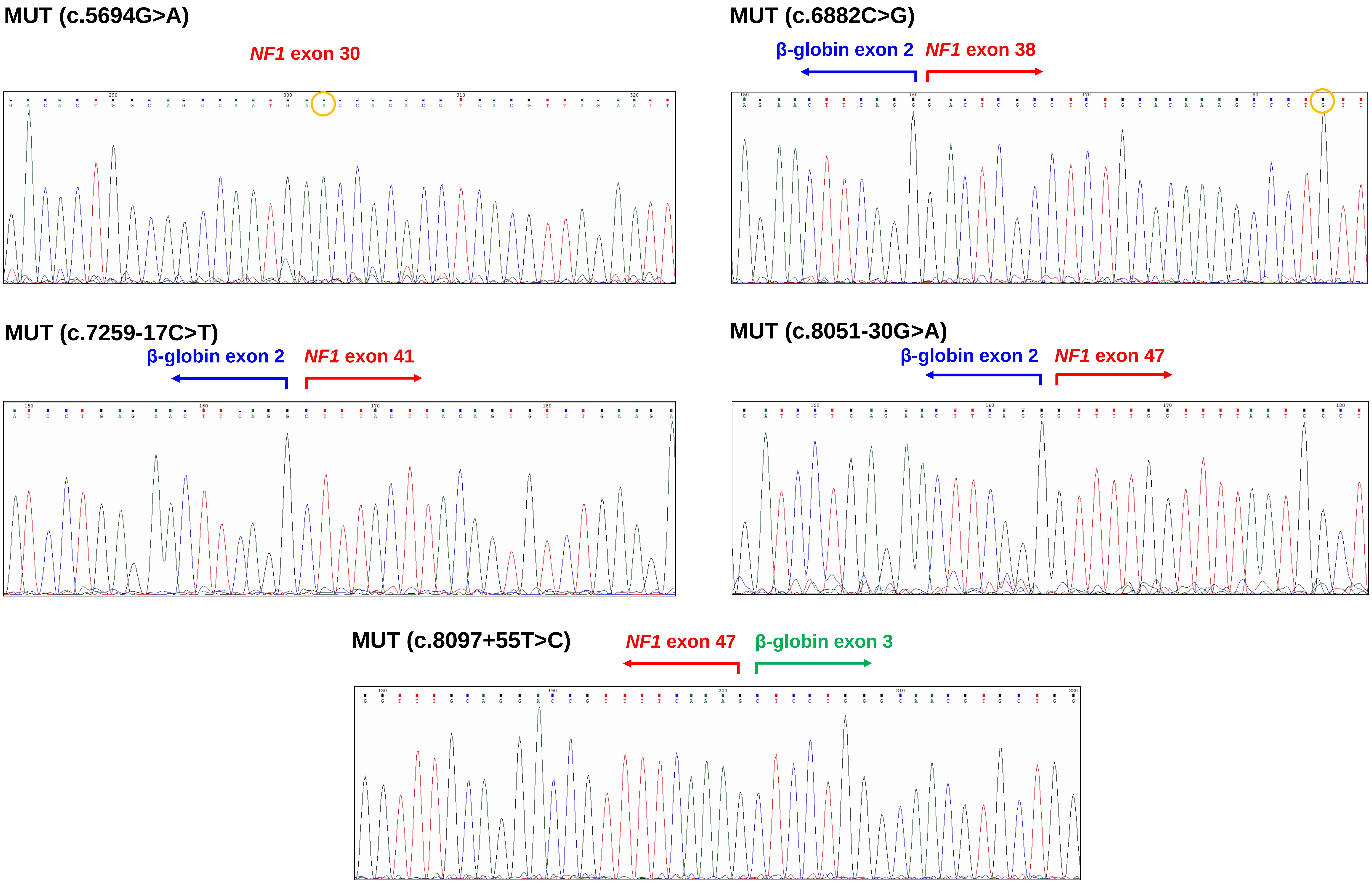
MUT (c.5694G>A)
NF1 exon 30
MUT (c.6882C>G)
β-globin exon 2
NF1 exon 38
MUT (c.7259-17C>T)
β-globin exon 2
NF1 exon 41
MUT (c.8051-30G>A)
β-globin exon 2
NF1 exon 47
MUT (c.8097+55T>C)
NF1 exon 47
β-globin exon 3

CANONICAL VARIANTS

c.1185+2T>G

WT

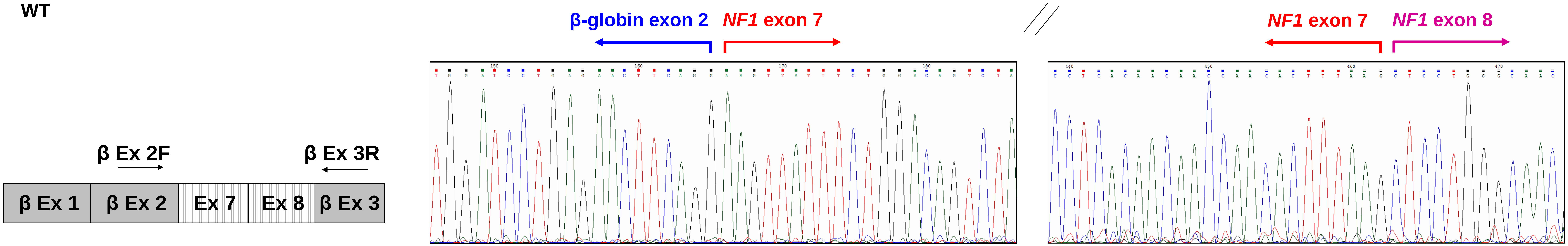

MUT

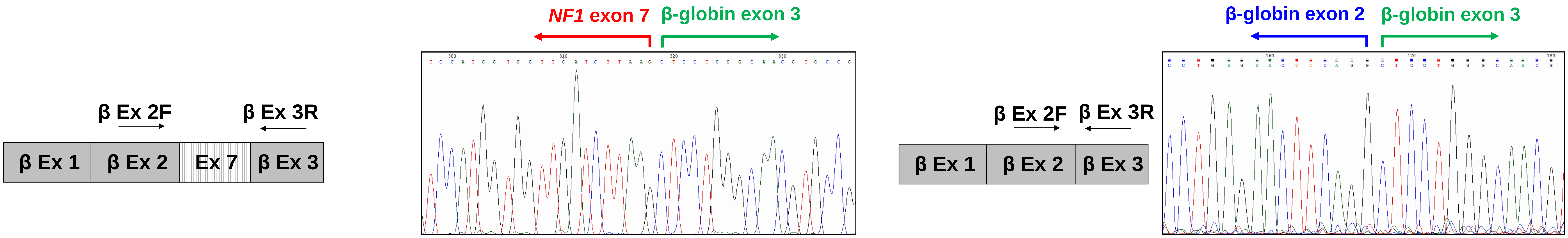

c.3496+1G>A

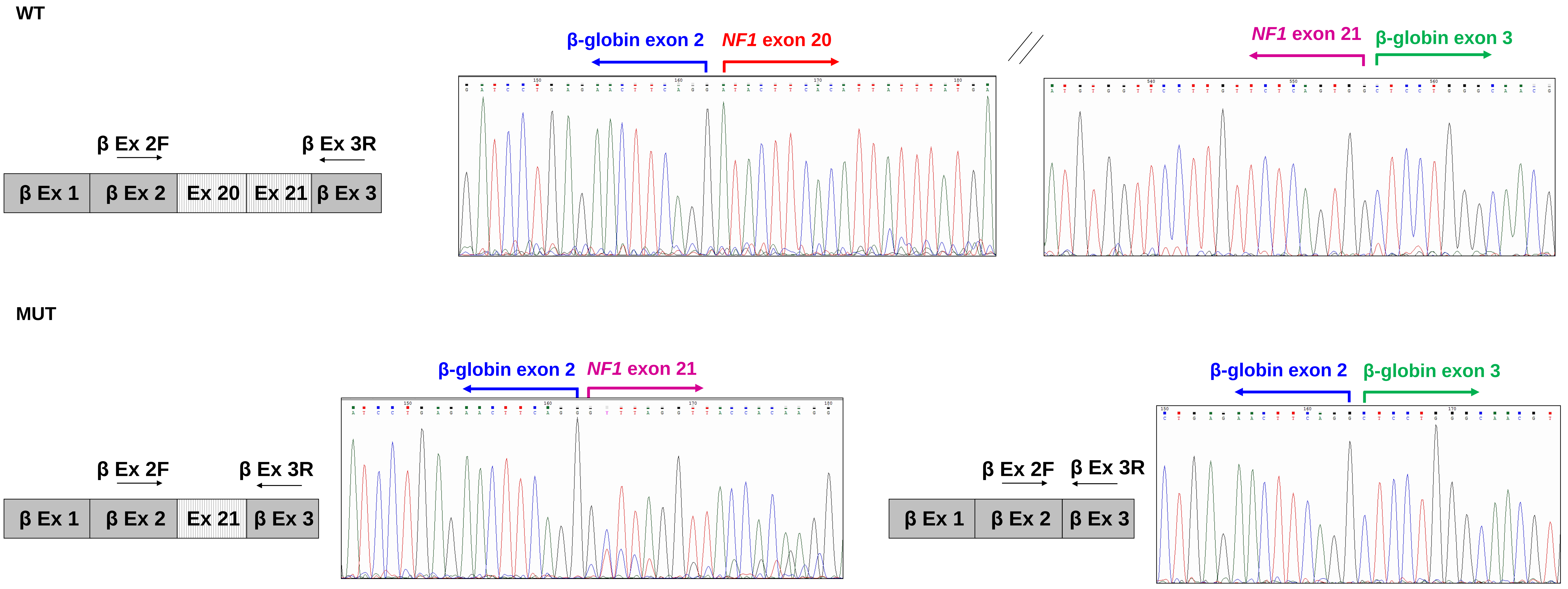

WT
β Ex 2F
β Ex 3R
β Ex 1
β Ex 2
Ex 20
Ex 21
β Ex 3
β-globin exon 2
NF1 exon 20
NF1 exon 21
β-globin exon 3
MUT
β Ex 2F
β Ex 3R
β Ex 1
β Ex 2
Ex 21
β Ex 3
β-globin exon 2
NF1 exon 21
β Ex 2F
β Ex 3R
β Ex 1
β Ex 2
β Ex 3
β-globin exon 2
β-globin exon 3

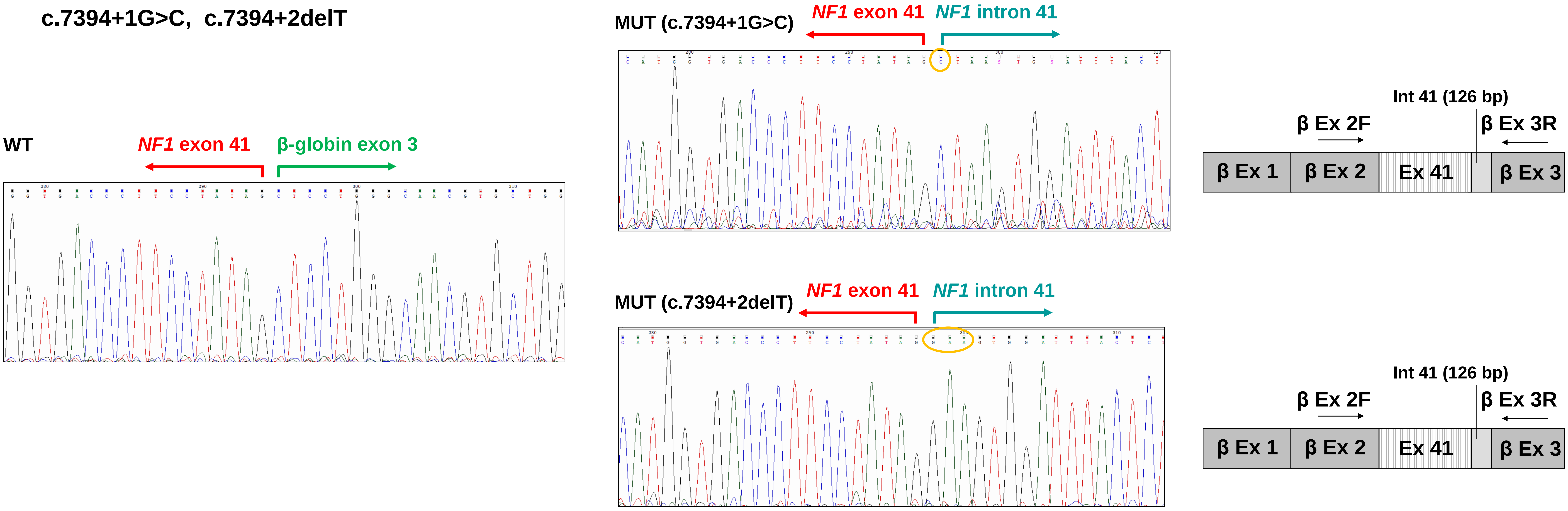

c.7394+1G>C, c.7394+2delT
WT
NF1 exon 41
β-globin exon 3
MUT (c.7394+1G>C)
NF1 exon 41
NF1 intron 41
MUT (c.7394+2delT)
NF1 exon 41
NF1 intron 41
Int 41 (126 bp)
β Ex 2F
β Ex 3R
β Ex 1
β Ex 2
Ex 41
β Ex 3
Int 41 (126 bp)
β Ex 2F
β Ex 3R
β Ex 1
β Ex 2
Ex 41
β Ex 3

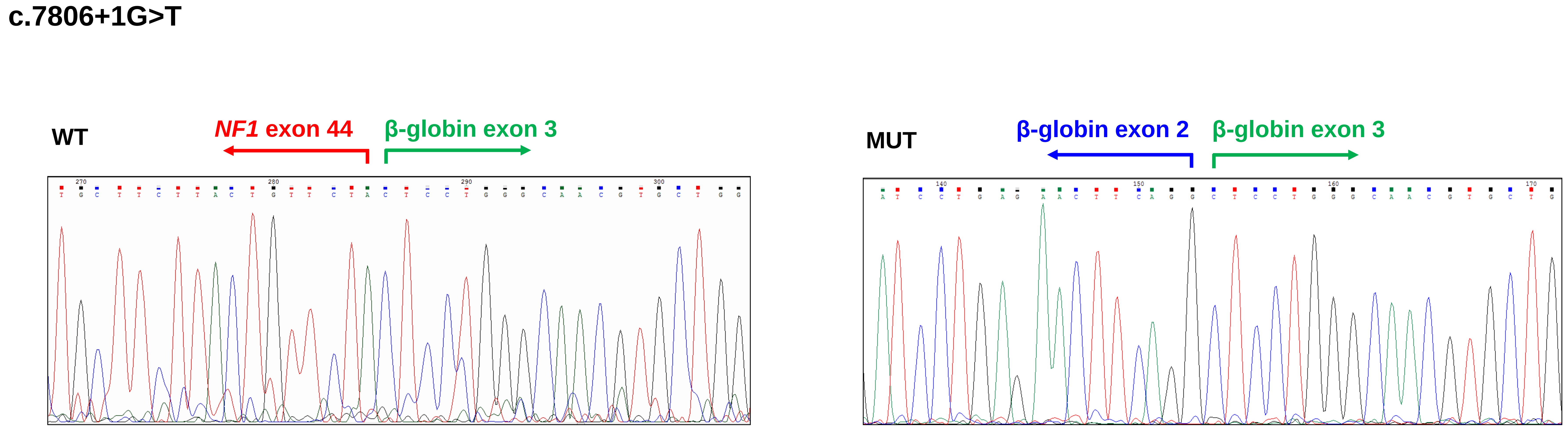

c.7806+1G>T
WT
NF1 exon 44
β-globin exon 3
MUT
β-globin exon 2
β-globin exon 3

NON-CANONICAL VARIANTS

c.278G>A

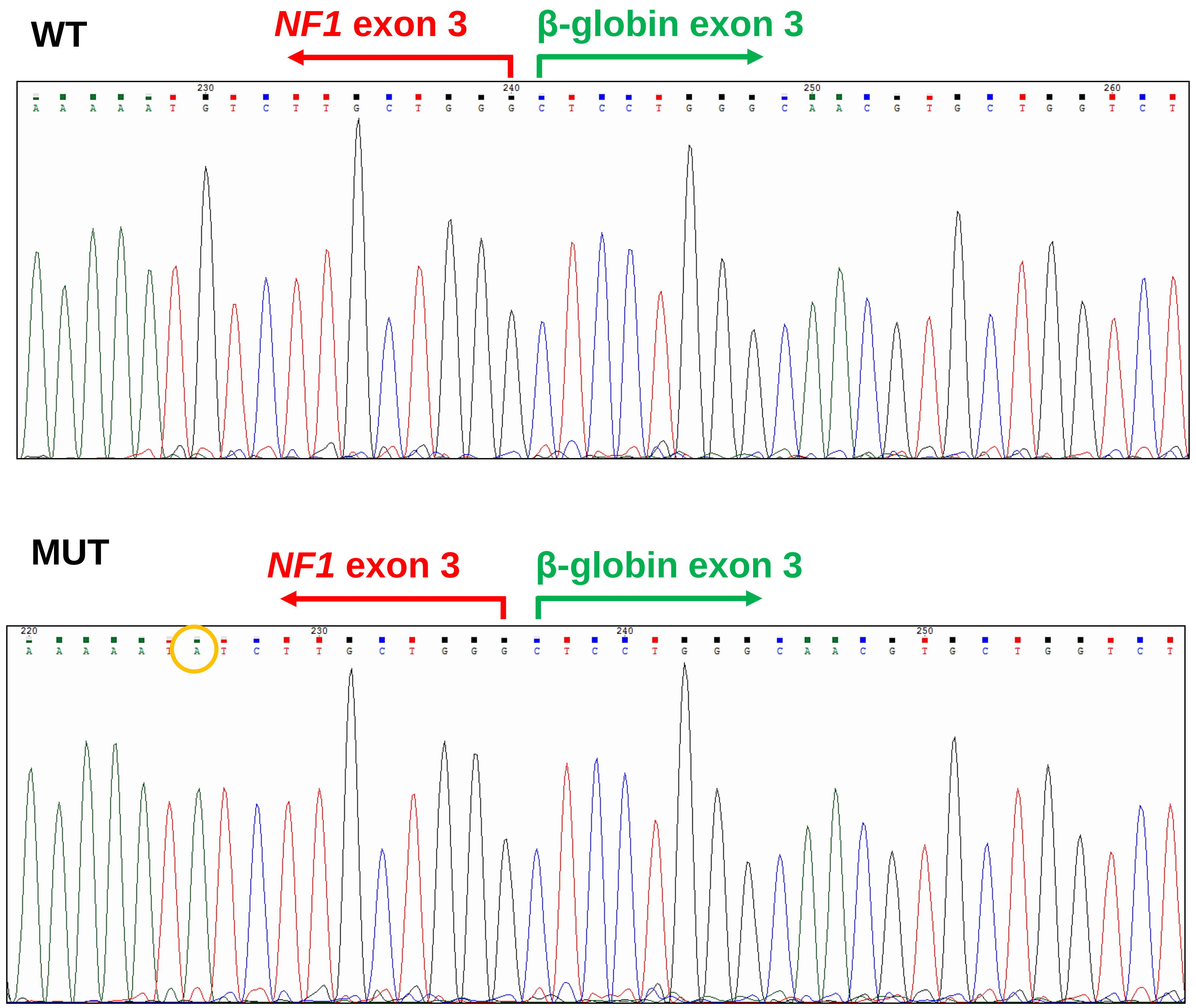

c.1466A>G

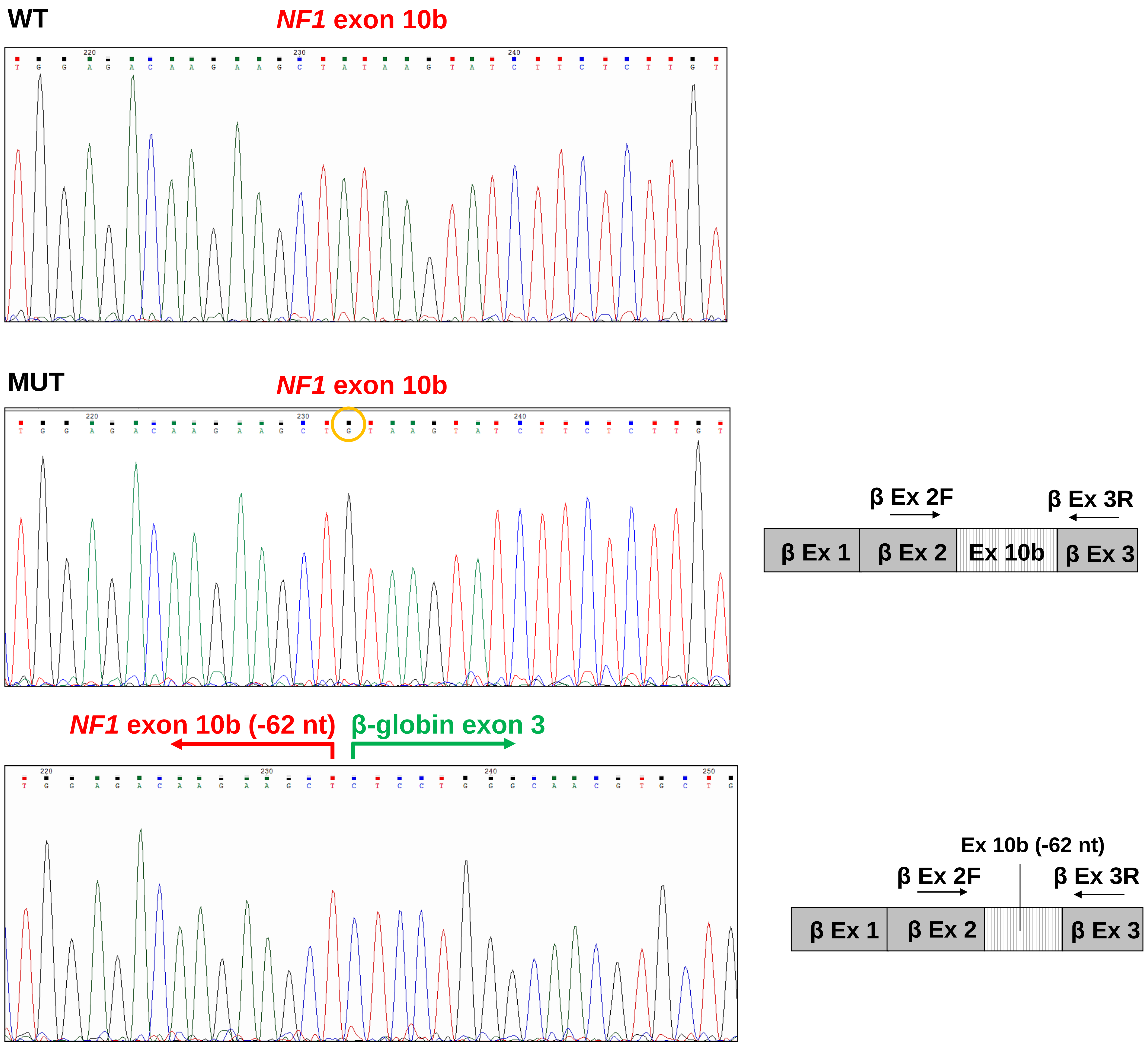

c.1527+1_1527+4delGTAA

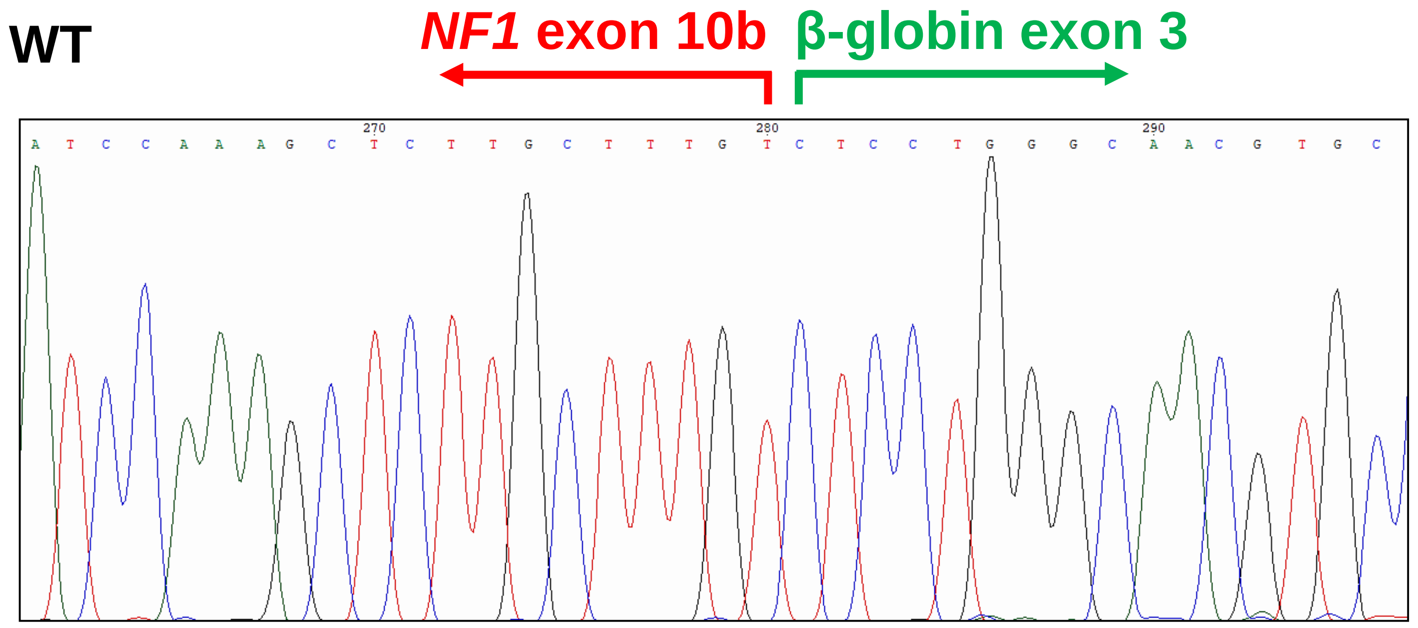

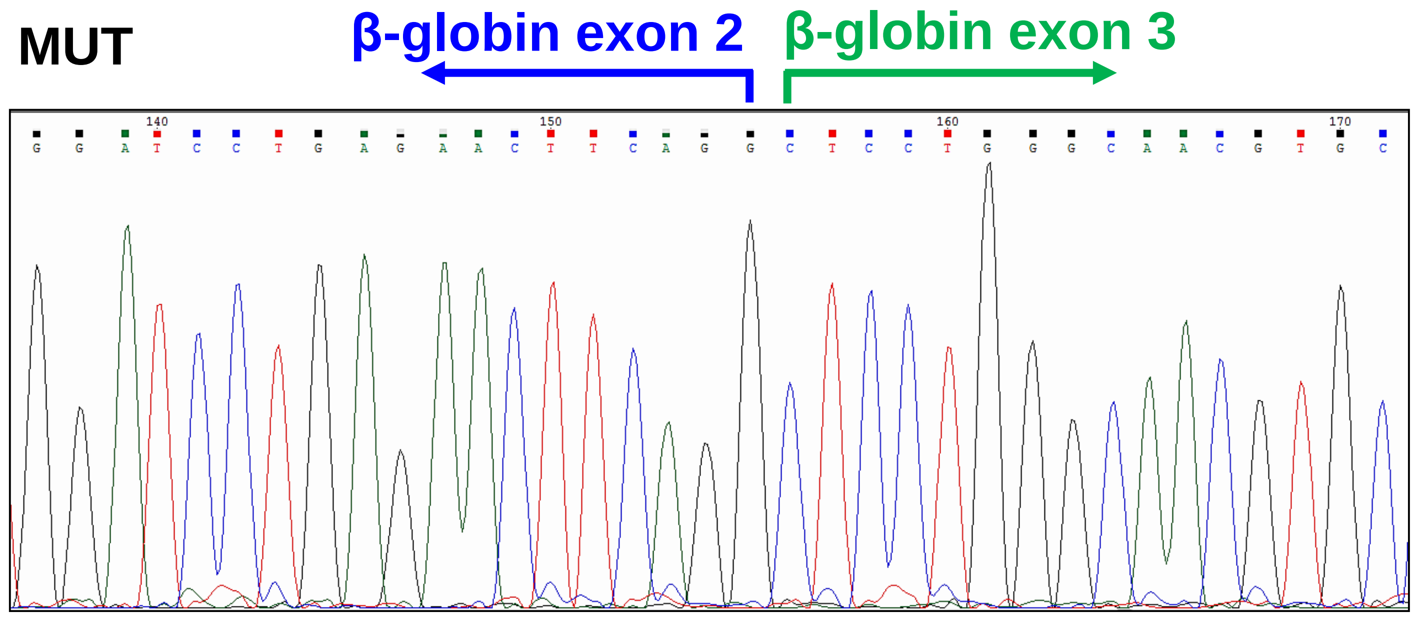

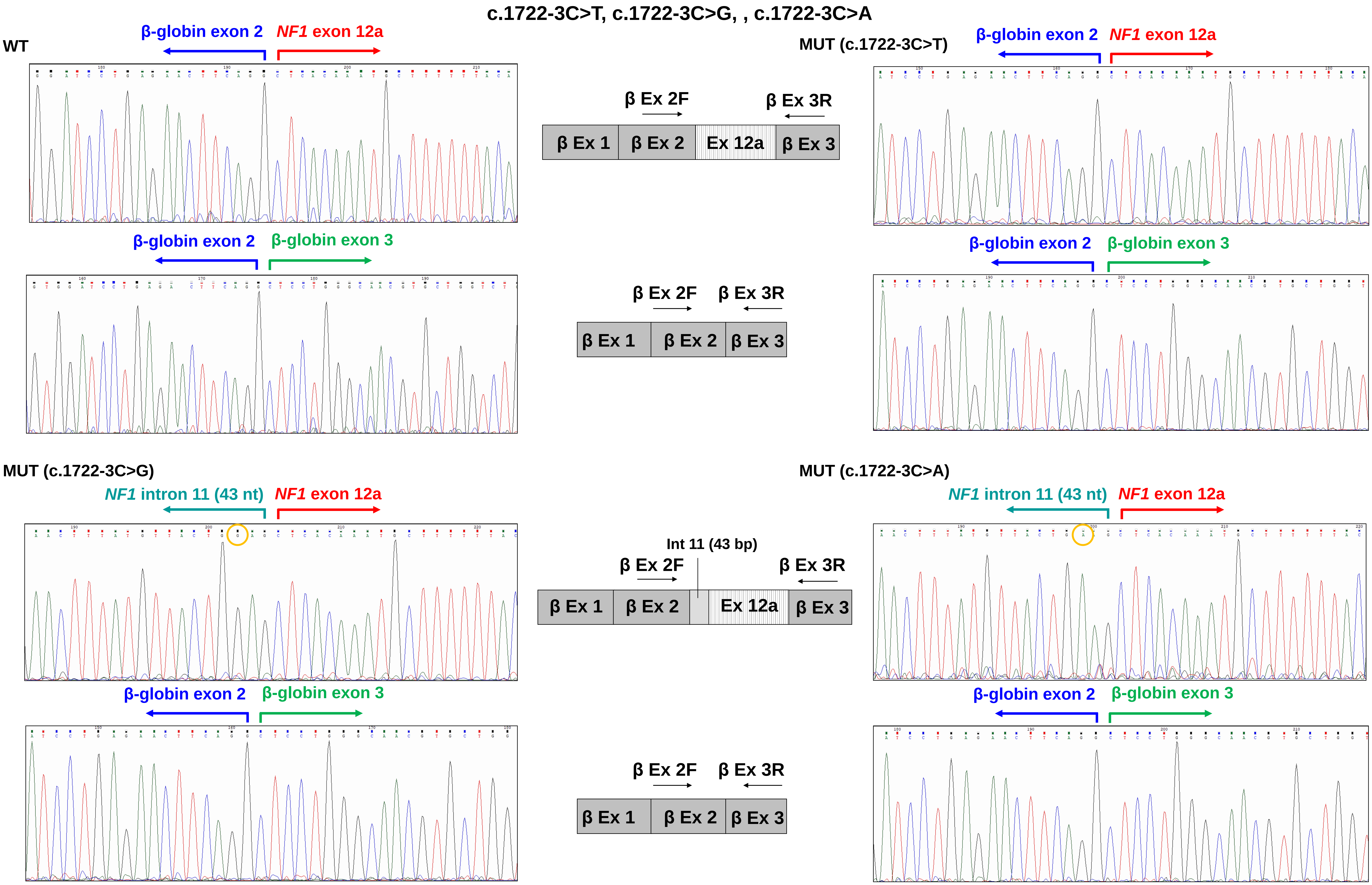
c.1722-3C>T, c.1722-3C>G, , c.1722-3C>A
WT
β-globin exon 2
NF1 exon 12a
β-globin exon 2
β-globin exon 3
β Ex 2F
β Ex 3R
β Ex 1
β Ex 2
Ex 12a
β Ex 3
β Ex 2F
β Ex 3R
β Ex 1
β Ex 2
β Ex 3
MUT (c.1722-3C>T)
β-globin exon 2
NF1 exon 12a
β-globin exon 2
β-globin exon 3
MUT (c.1722-3C>G)
NF1 intron 11 (43 nt)
NF1 exon 12a
β-globin exon 2
β-globin exon 3
Int 11 (43 bp)
β Ex 2F
β Ex 3R
β Ex 1
β Ex 2
Ex 12a
β Ex 3
β Ex 2F
β Ex 3R
β Ex 1
β Ex 2
β Ex 3
MUT (c.1722-3C>A)
NF1 intron 11 (43 nt)
NF1 exon 12a
β-globin exon 2
β-globin exon 3

c.2325+2dupT

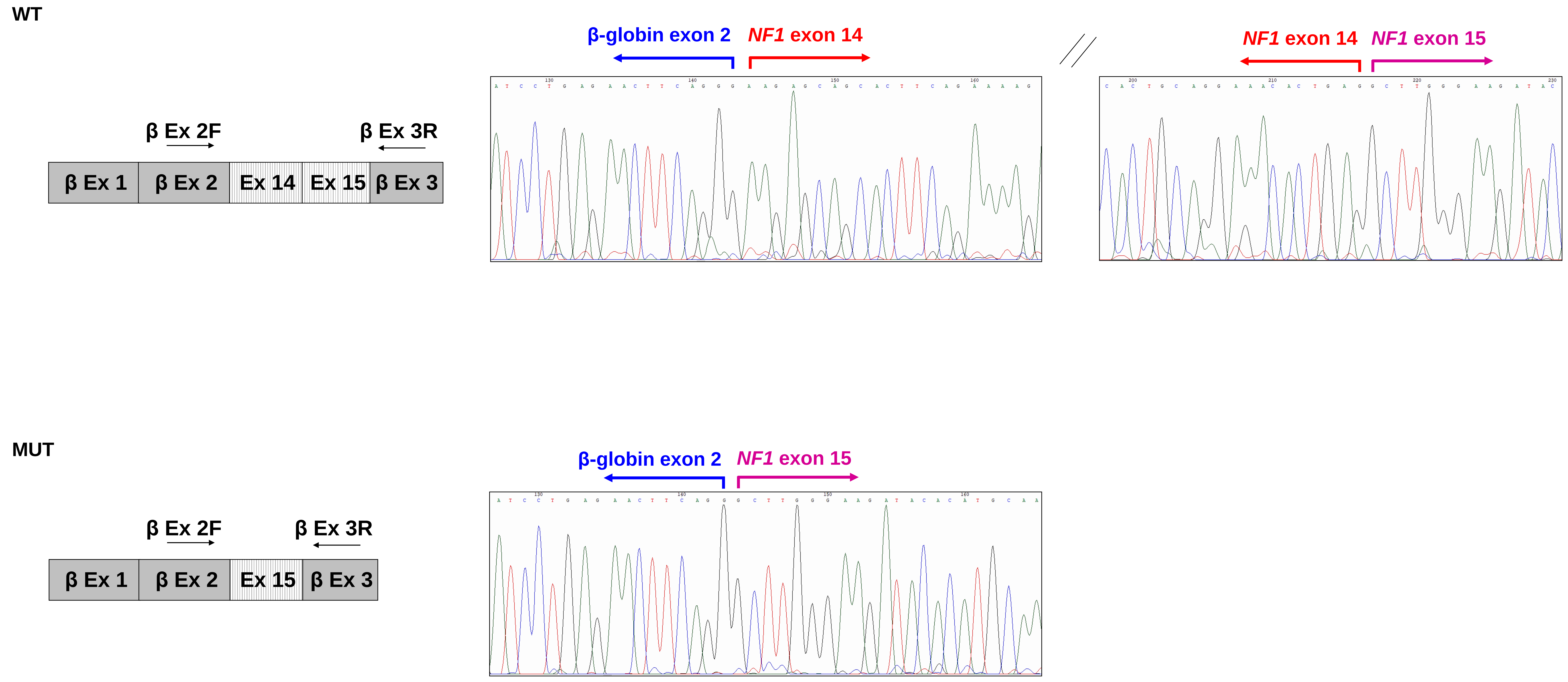

WT
β Ex 2F
β Ex 3R
β Ex 1
β Ex 2
Ex 14
Ex 15
β Ex 3
β-globin exon 2
NF1 exon 14
NF1 exon 14
NF1 exon 15
MUT
β Ex 2F
β Ex 3R
β Ex 1
β Ex 2
Ex 15
β Ex 3
β-globin exon 2
NF1 exon 15

c.3112A>G, c.3113+5G>A

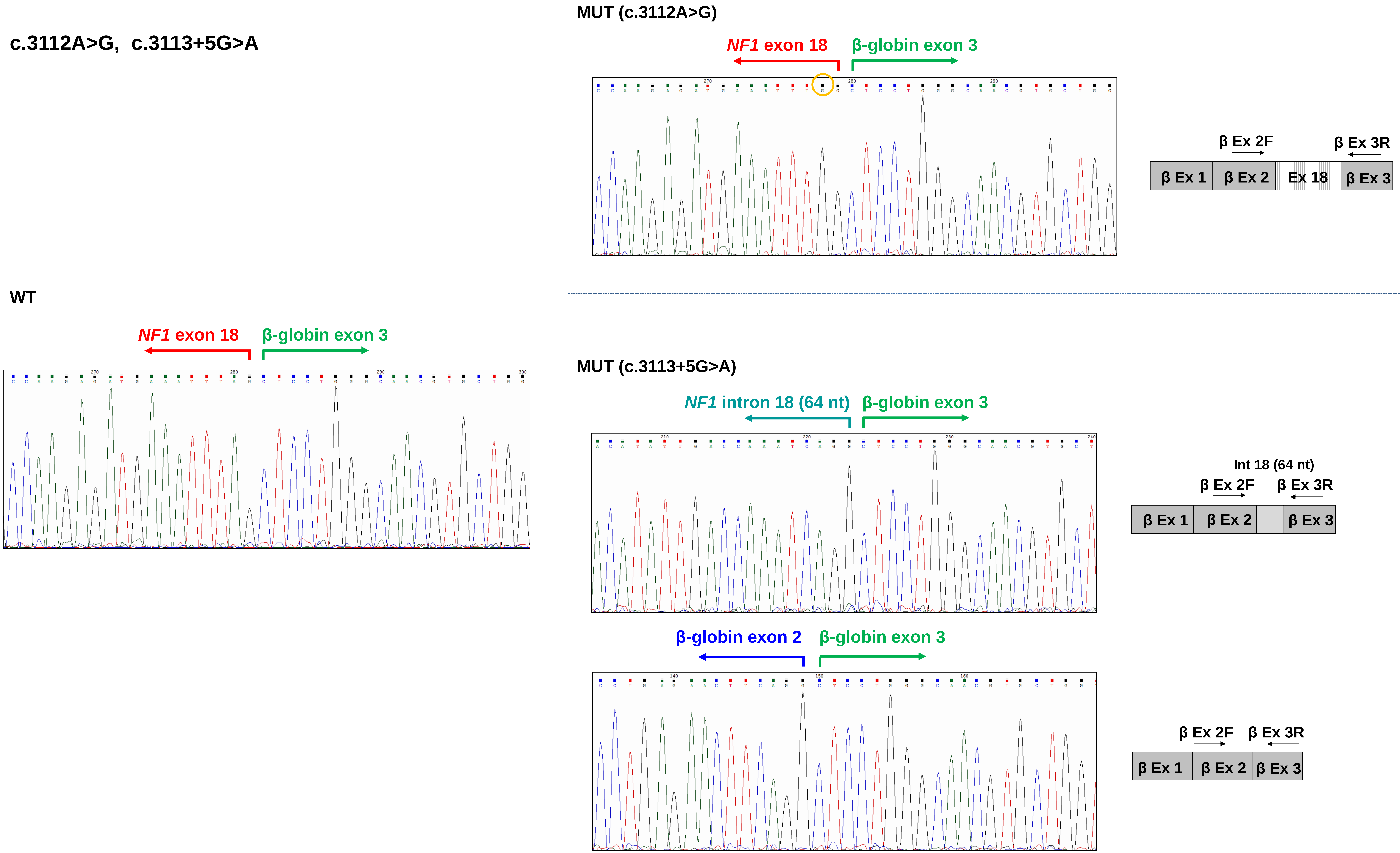

c.3496+3G>T, c.3496+5G>A

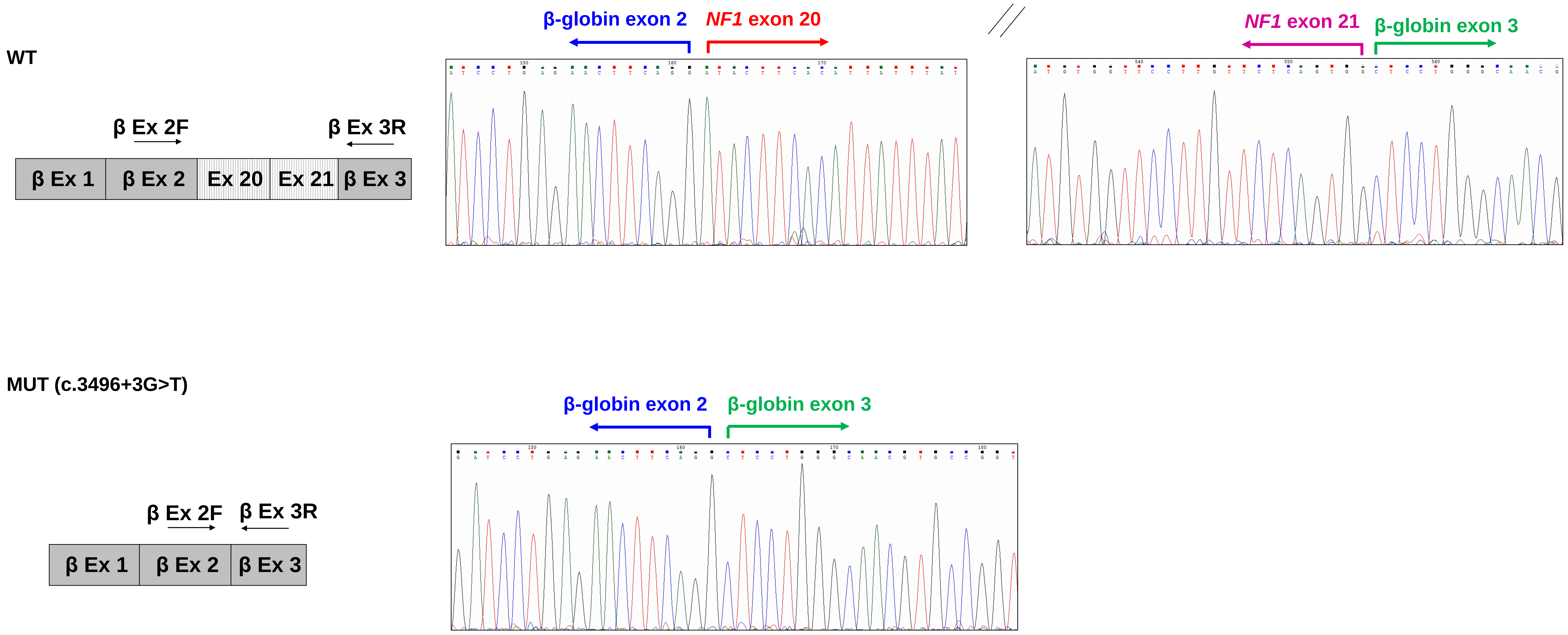

MUT (c.3496+5G>A)

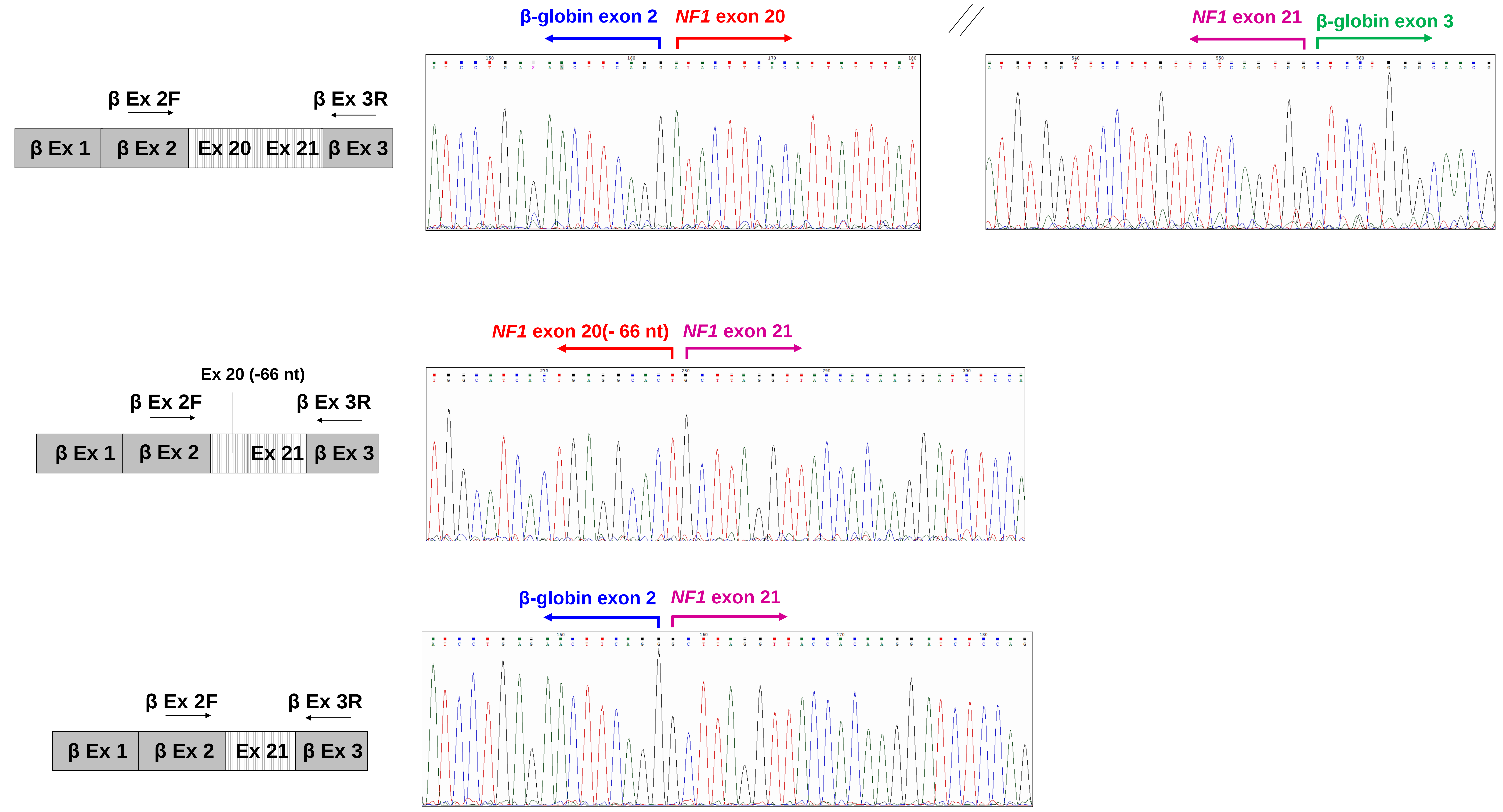

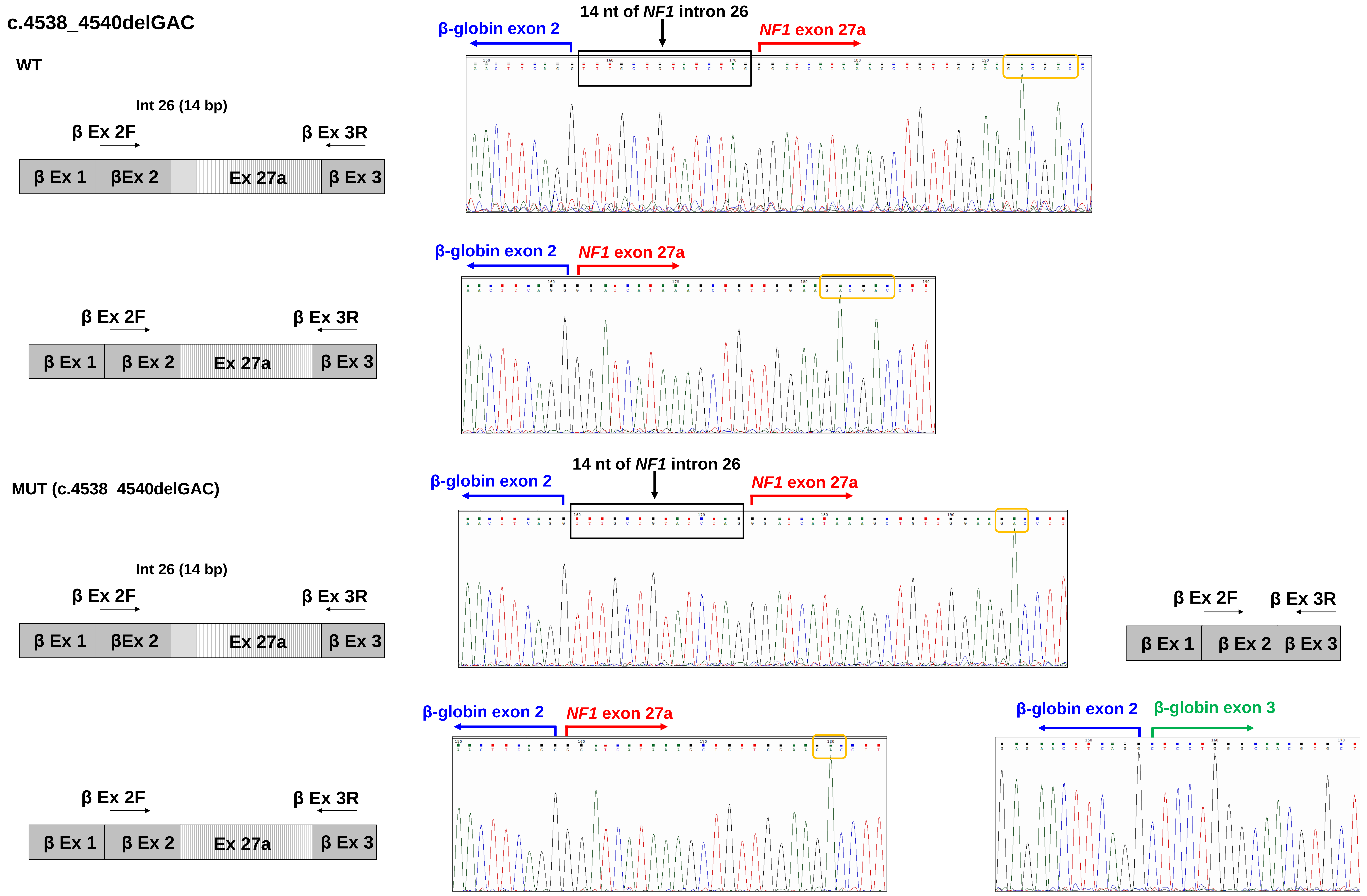
c.4538_4540delGAC
WT
Int 26 (14 bp)
β Ex 2F
β Ex 3R
β Ex 1
βEx 2
Ex 27a
β Ex 3
14 nt of NF1 intron 26
β-globin exon 2
NF1 exon 27a
β Ex 2F
β Ex 3R
β Ex 1
β Ex 2
Ex 27a
β Ex 3
β-globin exon 2
NF1 exon 27a
MUT (c.4538_4540delGAC)
Int 26 (14 bp)
β Ex 2F
β Ex 3R
β Ex 1
βEx 2
Ex 27a
β Ex 3
14 nt of NF1 intron 26
β-globin exon 2
NF1 exon 27a
β Ex 2F
β Ex 3R
β Ex 1
β Ex 2
β Ex 3
β Ex 2F
β Ex 3R
β Ex 1
β Ex 2
Ex 27a
β Ex 3
β-globin exon 2
NF1 exon 27a
β-globin exon 2
β-globin exon 3

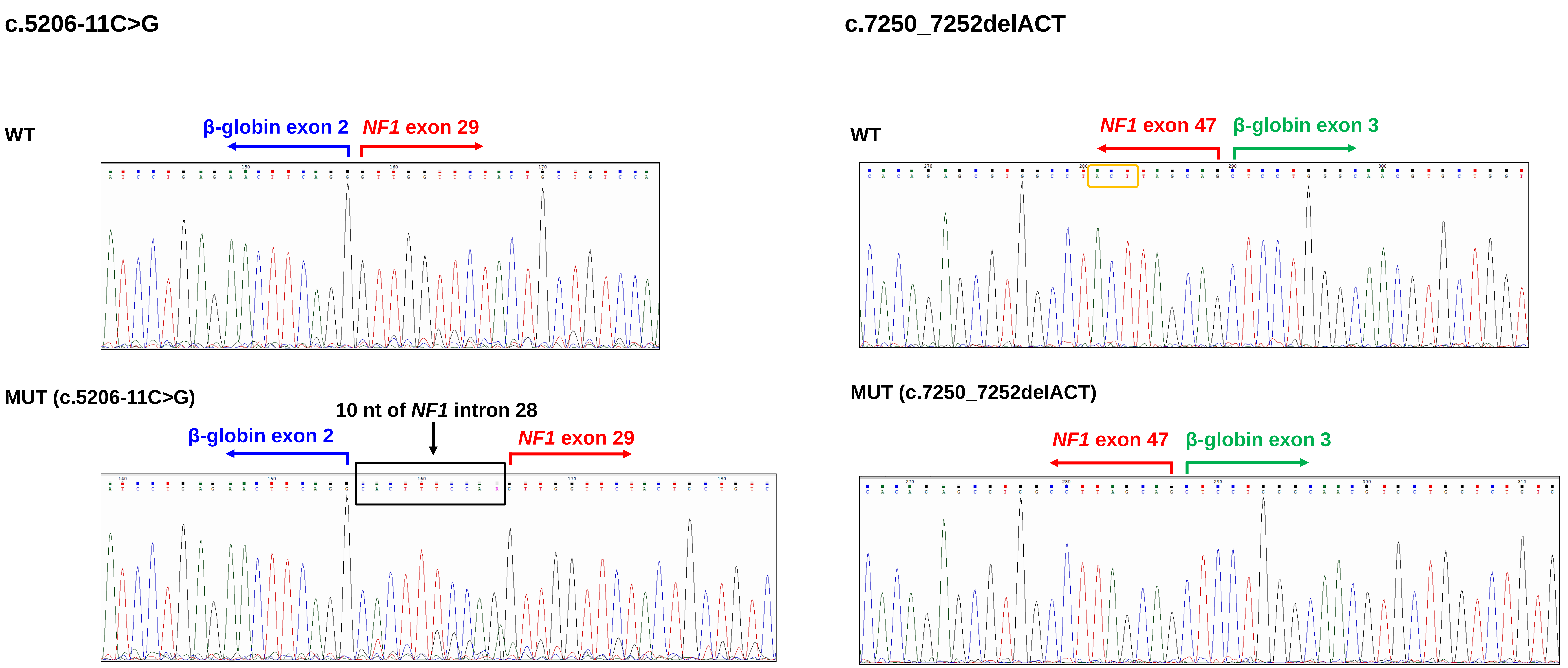

Figure S1. Sanger sequencing of the RT-PCR products obtained after transfection of HEK293 cells with the minigene construct harboring the indicated variant (MUT) or with the corresponding wild-type one (WT). For benign variants, that do not affect splicing, control sequences are not shown, while for canonical and non-canonical variants electropherograms obtained after transfection of both wild-type and mutated minigene constructs are depicted. When multiple transcripts were identified or when minigene constructs include more than one exon, a scheme of the resulting PCR products is represented next to each electropherogram. For the c.5206-11C>G substitution, both wild-type and mutated sequences show lower peaks corresponding to skipping of exon 29 (exon 37). Also for the c.3496+1G>A variant, the sequence corresponding to skipping of exon 20 shows also the presence of the product resulting from skipping of both exon 20 (exon 26) and 21 (exon 27).
Variants, if included within the electropherogram, are highlithed in yellow. nt, nucleotides.

Figure S2

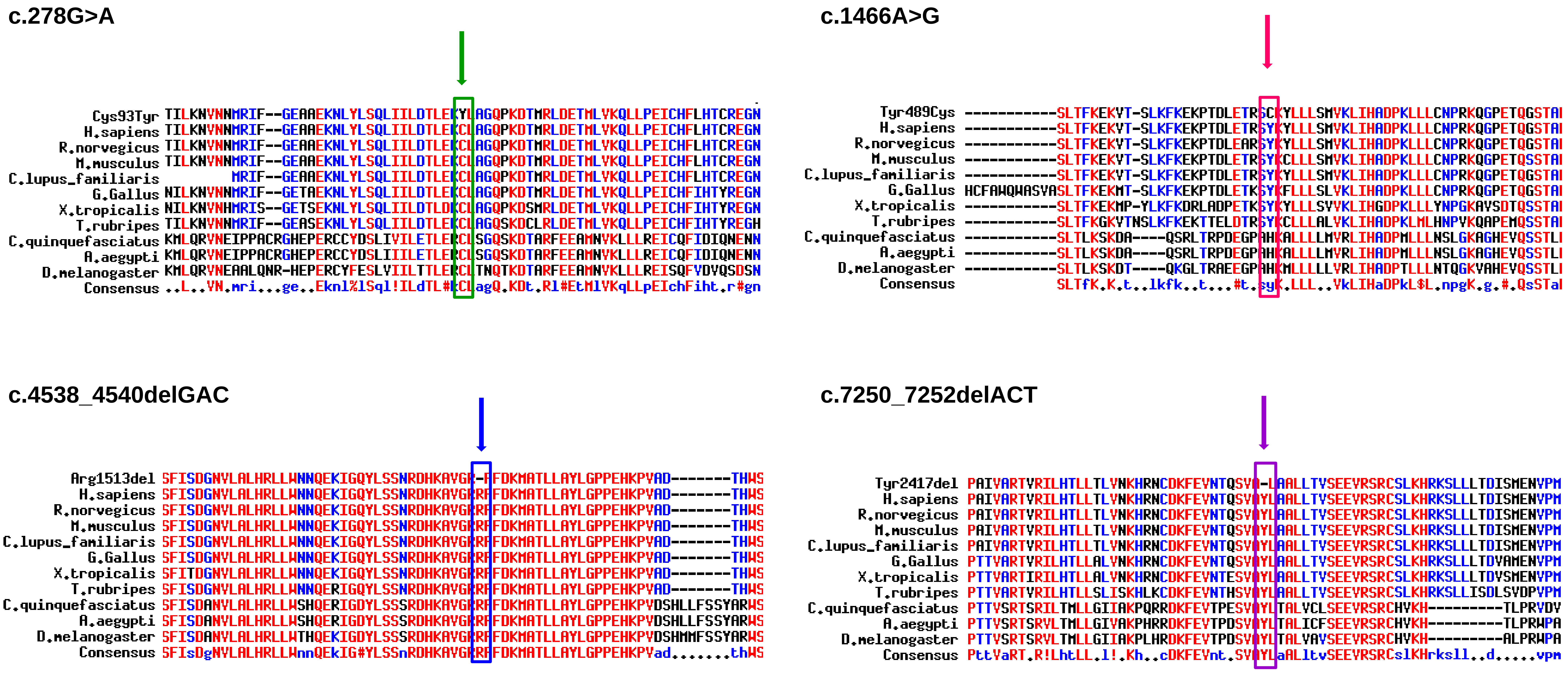
c.278G>A
c.1466A>G
Cys93Tyr
Tyr489Cys
H.sapiens
R.norvegicus
M.musculus
C.lupus_familiaris
G.Gallus
X.tropicalis
T.rubripes
C.quinquefasciatus
A.aegypti
D.melanogaster
Consensus
c.4538_4540delGAC
c.7250_7252delACT
Arg1513del
Tyr2417del

c.3496+5G>A

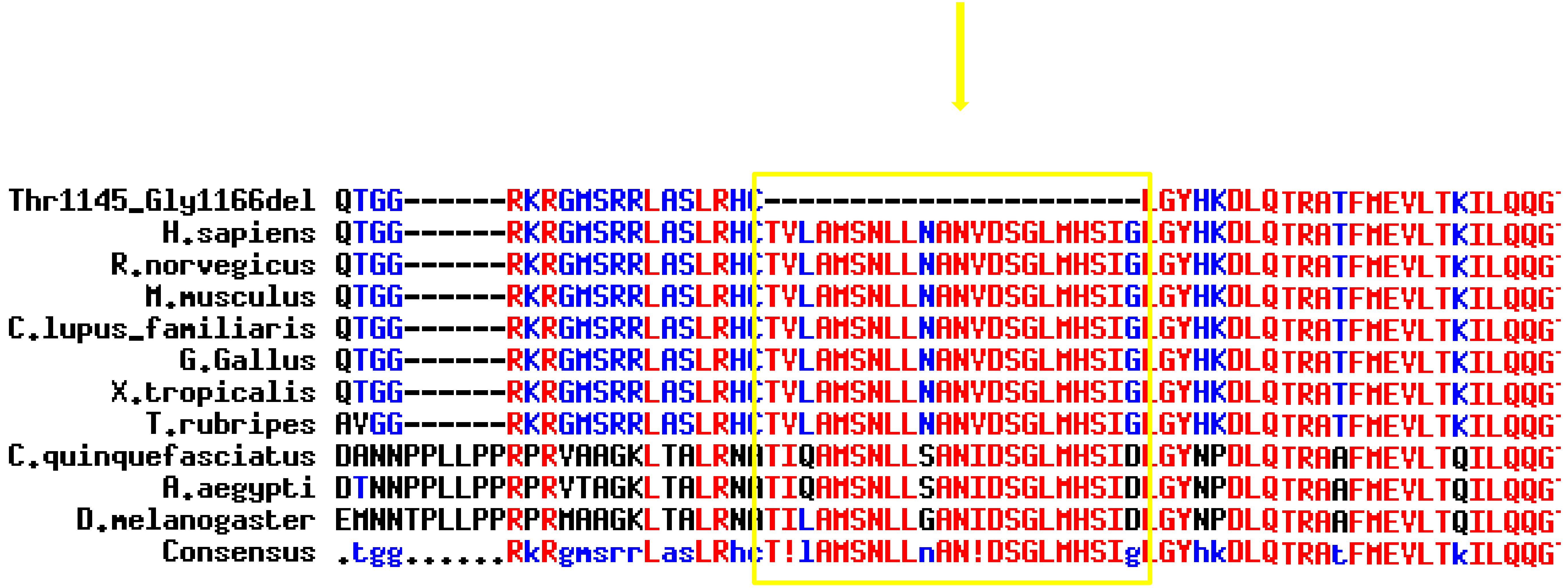

Figure S2. Multalin alignments of wild-type and mutant (p.Cys93Tyr, p.Tyr489Cys, p.Arg1513del, p.Tyr2417del and p.Thr1145_Gly1166del) human neurofibromin with orthologues of different species. A box and an arrow (green for p.Cys93Tyr, pink for p.Tyr489Cys, blue for p.Arg1513del and violet for p.Tyr2417del) point at each mutation, highlighting the level of conservation of the residue among species. The last alignment refers to the predicted mutant protein lacking 22 aminoacids of exon 20 (exon 26) (p.Thr1145_Gly1166del) resulting from the *inframe* loss of last 66 nucleotides of exon 20, a splicing product identified by the minigene assay for the c.3496+5G>A variant. A yellow box and arrow highlight that this deleted portion of neurofibromin is highly conserved among different species.

Figure S3

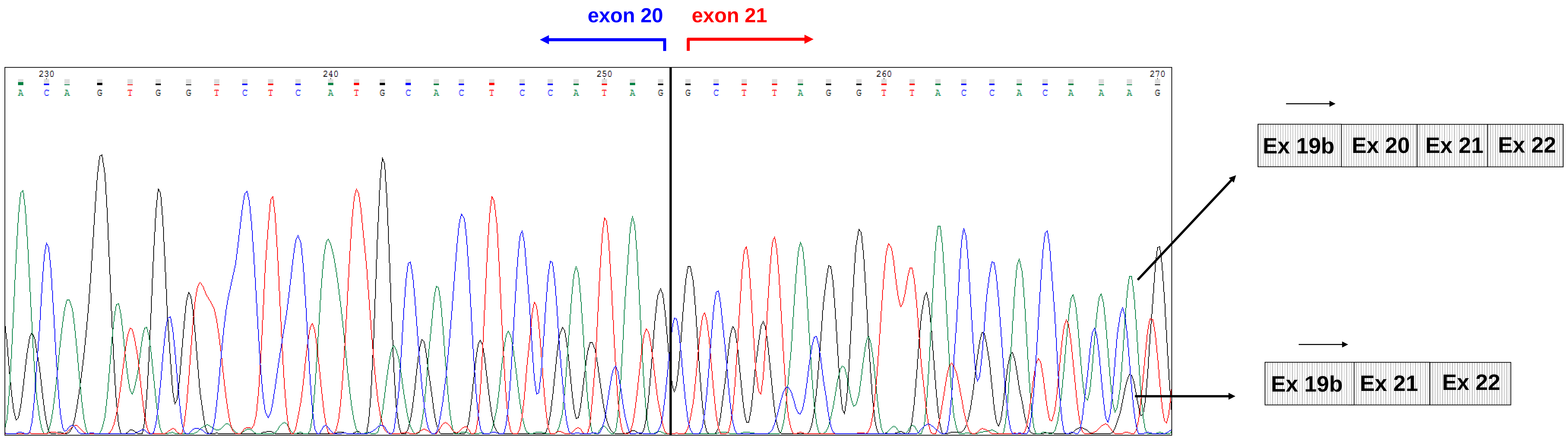

Figure S3. cDNA analysis of *NF1* in the patient harboring the c.3496+5G>A variant. The whole coding region of *NF1* was amplified into five overlapping 2 kb amplicons. The electropherogram of amplicon 3 showed the presence of two transcripts, differing for the presence of exon 20 (exon 26), as illustrated by the schematic representation next to the Sanger sequence. The arrow indicates the primer used to sequence cDNA.

Figure S4

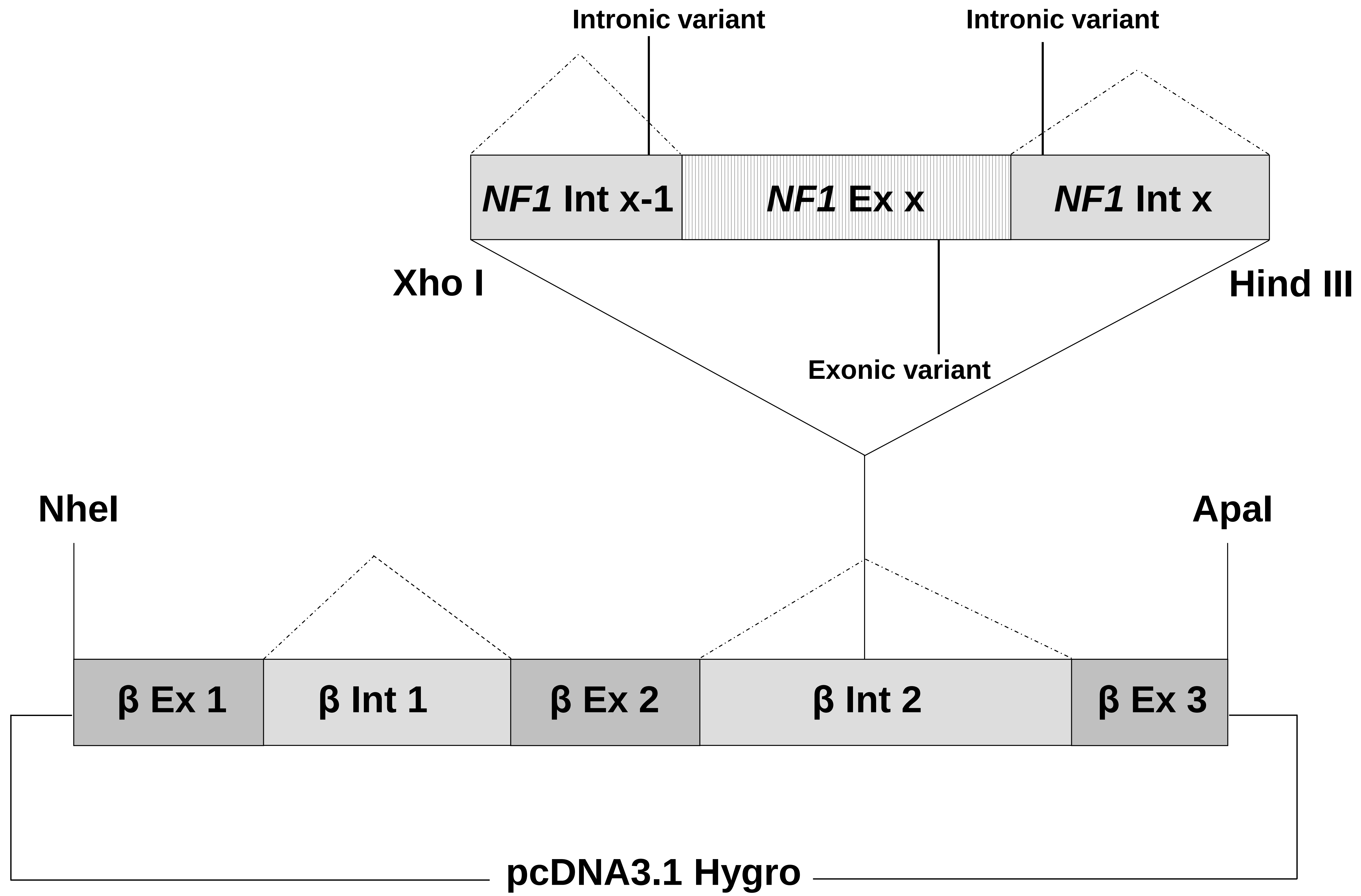

Figure S4. A schematic representation of the hybrid minigene construct used in the experiments. PCR fragments including the exon (Ex) adjacent to each *NF1* variant and at least 100 bp of the upstream and downstream introns (Int) were amplified from patients' genomic DNA and cloned into the β-globin vector, which was obtained by the cloning of the β-globin gene inside a pcDNA™3.1/Hygro(+) vector (ThermoFischer Scientific). β, β-globin.
